# Birth weight associations with DNA methylation differences in an adult population

**DOI:** 10.1080/15592294.2020.1827713

**Published:** 2020-10-20

**Authors:** Rebecca A. Madden, Daniel L. McCartney, Rosie M. Walker, Robert F. Hillary, Mairead L. Bermingham, Konrad Rawlik, Stewart W. Morris, Archie Campbell, David J. Porteous, Ian J. Deary, Kathryn L. Evans, Jonathan Hafferty, Andrew M. McIntosh, Riccardo E. Marioni

**Affiliations:** a Division of Psychiatry, University of Edinburgh, Royal Edinburgh Hospital, Edinburgh, UK; b Centre for Genomic and Experimental Medicine, Institute of Genetics and Molecular Medicine, University of Edinburgh, Edinburgh, UK; c Centre for Cognitive Ageing and Cognitive Epidemiology, University of Edinburgh, Edinburgh, UK; d The Roslin Institute, Royal (Dick) School of Veterinary Studies, University of Edinburgh, Midlothian, UK; e Department of Psychology, University of Edinburgh, Edinburgh, UK

**Keywords:** DNA methylation, Generation Scotland, birth weight, depression, EWAS

## Abstract

The Developmental Origins of Health and Disease (DOHaD) theory predicts that prenatal and early life events shape adult health outcomes. Birth weight is a useful indicator of the foetal experience and has been associated with multiple adult health outcomes. DNA methylation (DNAm) is one plausible mechanism behind the relationship of birth weight to adult health. Through data linkage between Generation Scotland and historic Scottish birth cohorts, and birth records held through the NHS Information and Statistics Division, a sample of 1,757 individuals with available birth weight and DNAm data was derived. Epigenome-wide association studies (EWAS) were performed in two independently generated DNAm subgroups (n_Set1_ = 1,395, n_Set2_ = 362), relating adult DNAm from whole blood to birth weight. Meta-analysis yielded one genome-wide significant CpG site (p = 5.97x10^−9^), cg00966482. There was minimal evidence for attenuation of the effect sizes for the lead loci upon adjustment for numerous potential confounder variables (body mass index, educational attainment, and socioeconomic status). Associations between birth weight and epigenetic measures of biological age were also assessed. Associations between lower birth weight and higher Grim Age acceleration (p_(FDR)_ = 3.6x10^−3^) and shorter DNAm-derived telomere length (p_(FDR)_ = 1.7x10^−3^) are described, although results for three other epigenetic clocks were null. Our results provide support for an association between birth weight and DNAm both locally at one CpG site, and globally via biological ageing estimates.

## Introduction

The Developmental Origins of Health and Disease theory (DOHaD) states that through developmental plasticity, the foetal experience can permanently influence adult health [1]. The theory’s main proponent, David Barker, originally relied on birth weight as an index of foetal nutrition – an assumption that has been contested by the awareness that multiple factors can influence birth weight [2]. Maternal stress, illness, and socioeconomic status [3–5] are among modifiable influences over offspring birth weight. In addition, foetal genetics and maternal genetics both influence birthweight, the latter acting through the intrauterine environment [6,7]. Thus, birth weight can be seen as an index of the general foetal experience.

There is strong, well-replicated evidence for an association between birth weight and adult health. There is particularly consistent evidence for associations between low birth weight and poor cardiovascular outcomes, such as heart disease, type II diabetes, stroke, and hypertension [8–11], as well as poorer cognitive ability and a raised risk for mood disorders [12,13]. Birth weight is clinically conceptualized as ‘low’ below 2.5 kg [14], however it can also be analysed on a continuous scale. These associations are found after accounting for adult lifestyle factors, such as smoking and body mass index (BMI), indicating a residual association between birth weight and adult health outcomes.

Prenatal factors affect the foetus in its highly plastic state, giving rise to birth weight variability, and also to developmental changes which permanently affect the function and health of organs and systems [1]. Given the genetic and environmental contributions to birth weight variability, epigenetic modifications such as DNA methylation (DNAm) might provide insights into the pathways and mechanisms through which these associations with health become manifest. DNAm is typically characterized by the addition of a methyl group to cytosine nucleotides in the context of cytosine-guanine (CpG) dinucleotides. DNAm changes are linked to the regulation of gene expression, providing a possible mechanism through which environmental influences may have lasting biological effects [15]. Therefore, DNAm is one putative mechanism through which developmental experience may relate to adult health.

Birth weight associations with DNAm have been previously described in neonatal blood [16–18] and during childhood [19], seeming to diminish into adolescence and beyond [16,18]. The established association between differential DNAm and birth weight in these highly plastic early years raises the possibility of a direct effect of transcriptional up- or down-regulation on the development of organs and tissues [20]. This is one way in which DNAm may mediate the association between birth weight and adult health. It is possible, however, that examining only differential DNAm patterns which persist from birth into the later years ignores the full adult epigenome which, despite having changed since birth, may provide information on a relationship between birth weight and downstream health outcomes nevertheless. Many of the health phenotypes associated with birthweight present in adulthood, so the question remains whether we can see biological marks of these associations laid down in the methylome. It has, for instance, been demonstrated that prenatal famine exposure is associated with differential methylation in late adulthood [21]. It is therefore plausible that other prenatal factors may continue to influence DNAm into adulthood. While none of the longitudinal DNAm analysis studies have yet identified any epigenome-wide significant evidence of persistent CpG methylation in adulthood, there are some associations which do have p < 1x10^−5^ [18] but do not withstand Bonferroni correction for multiple testing. This may be tentatively interpreted as evidence that the adult epigenome holds information relating to birth weight, so a full, unbiased scan of the adult methylome seems a logical step. We would hope for this agnostic approach to confirm any genuine DNAm relationships in loci identified in other samples at birth, as well as revealing any new sites which might provide information on biological pathways through which the birth weight/health relationship is operating.

In addition to the stand-alone importance of discovering DNAm associations with birth weight in adults, recent work has revealed the utility of so-called ‘epigenetic clocks’ in demonstrating elements of vulnerability to disease and mortality [22]. These clocks attempt to calculate an individual’s biological age, and the residual of biological age regressed on chronological age (i.e. age acceleration) can be used to indicate health vulnerabilities. The earliest versions of epigenetic clocks, such as the pan-tissue Horvath clock [23] and the leukocyte-based Hannum clock [24], were trained simply on chronological age. The second generation of these clocks added clinical information on top of chronological age, to improve the biological relevance of the measure. The PhenoAge clock is of this second generation, it is based on clinical biomarkers known to associate with mortality [25], as is GrimAge (a predictor combining DNAm proxies for smoking and seven plasma proteins and trained on time-to-death [26]). Finally, a DNAm-derived estimate of telomere length outperforms phenotypic measures of telomere length [27], with validated utility in predicting lifespan and other aspects of health [27,28]. DNAm associations with birthweight, together with epigenetic clock analyses, have the potential to describe the known associations between birthweight and many aspects of whole-body health in adulthood.

Here, we report an Epigenome-Wide Association Study (EWAS) of birthweight using whole blood DNA derived in an adult sample, and associations between birth weight and five epigenetic clocks. While previous work has attempted to find persistent DNAm relationships to birth weight from infancy to adulthood [16,18], none have performed an agnostic, genome-wide study of DNAm in adults. We, therefore, hypothesize that while birth weight associated DNAm patterns may change over time, differences will exist in adulthood.

## Materials and methods

**Figure 1.**
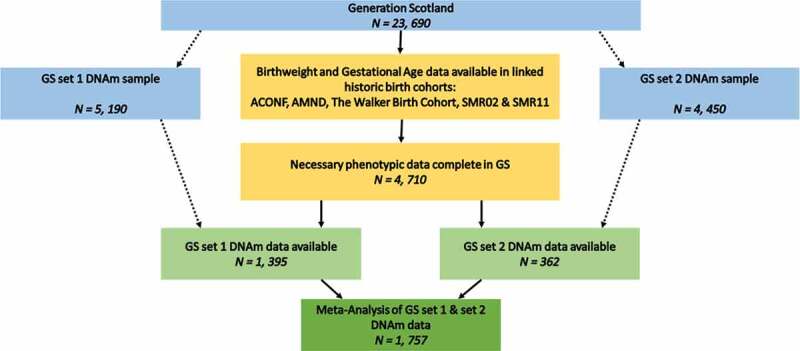
Inclusion flow diagram detailing the selection of samples for the current study

This study was performed on a subgroup of the Generation Scotland (GS) cohort whose birthweight data were collected at parturition in historical cohort studies. Data linkage strategies were employed to ascertain birthweight in grams from those historical cohorts. A subgroup of these individuals for whom DNA methylation data were processed formed the sample for EWAS and epigenetic clock analyses (Figure 1).

### Generation Scotland and other cohorts

Generation Scotland (GS) is a Scottish family-based cohort n = 23,690 [29]. Data were collected from participants between 2006 and 2011. GS is a deeply-phenotyped cohort, allowing examination of many aspects of adult health, alongside genomic and biometric measures. In addition, 98% of GS participants gave informed consent for data linkage to routinely collected health data and to information from other Scottish population cohort studies, both current and historical. These include several with neonatal and maternity information: the Aberdeen Children of the 1950s [30]; the Aberdeen Maternity and Neonatal Databank [31]; the Walker Birth Cohort [32]; and the Scottish Morbidity Records ([33], SMR02 – the Maternity Inpatient and Day Case record, and SMR11 – the Neonatal Inpatient dataset). Birth weight in grams, alongside gestational age at birth and twin information, was collated from these sources and linked to adult GS records for 4,710 participants (Supplementary File 1, Figure 1). Birth weight data derived from health records taken at birth has been shown to improve slightly on self-reported birth weight, which is often used in population cohorts [34].

### Statistical analyses

All analyses were conducted in R version 3.5.1 [35].

To control for the known effects of gestational age and sex on birthweight [36], we considered the scaled residuals (mean=0, SD=1) from a regression model in place of raw birth weight throughout:

$$Birth\;weight\left(g\right){\;}_{\;}sex+gestational\;age$$

These are referred to as ‘birth weight residuals’ hereafter.

### Epigenome-wide association study

Peripheral whole blood genome-wide DNA methylation was profiled in Generation Scotland in two different sets of ~5,000 samples. First, data were generated for 5,190 individuals, using the Illumina HumanMethylationEPIC BeadChip (Illumina Inc., San Diego, CA). Quality control and normalization were carried out as described elsewhere ([37,38]; **Supplementary File 2**). Birth weight and gestational age information were available for n = 1,395 of this methylation set (Figure 1), which will hereafter be referred to as ‘set 1’.

The second set of DNAm data used a near identical protocol (**Supplementary File 2**), creating an independent set of methylation data for an additional 4,450 GS participants. In this replication sample, a further 362 participants with both birth weight and gestational age information were used, hereafter called 'set 2'.

The birth weight residuals described above were used in the EWAS model, which was run using the ‘limma’ package in R (empirical Bayes moderated t-statistics). The set 1 EWAS model used CpGs corrected for relatedness (**Supplementary File 2**), as the first batch of DNAm data were collected on related individuals:

CpG ~ birth weight residuals + age + sex + smoking [ever/never] + smoking pack years + 20 methylation PCs

The 20 methylation PCs were included to try and eliminate unmeasured confounders in the DNAm data. Additional covariates (estimated white blood cell proportions – CD4T, CD8T, Granulocytes, BCells, Natural Killer cells – and methylation batch), which were regressed out during the relatedness pre-correction for the set 1 dataset, were also included in the set 2 dataset of unrelated individuals.

EWAS findings are considered epigenome-wide significant if p < 3.6x10^−8^ [39].

### Meta-analysis of set 1 and set 2 samples

An inverse variance-weighted meta-analysis of the set 1 and set 2 EWASs was performed using the METAL software package. Summary statistics from the set 1, set 2, and meta-analysis EWASs are available at https://doi.org/10.7488/ds/2876.

### Subsample analysis excluding preterm births

To account for the possible differences between preterm- and term-born infants, we re-ran the analyses detailed above on a subsample excluding those with gestational age <37 weeks. This is in line with the World Health Organization’s guidelines on preterm birth which states that birth before 37 completed weeks of gestation should be defined as preterm [40]. The total subsample had n_Set1_ = 1,346, n_Set2_ = 351, resulting in a total n_Meta_ = 1,697. By contrast, the full sample discussed in this report comprises both preterm and term born individuals.

### Sensitivity analysis

As a sensitivity analysis, the EWAS meta-analysis was re-examined, fitting additional covariates individually, and together in a fully-adjusted model. Covariates were selected to account for additional lifestyle factors which may associate with DNAm or birth weight. These variables could represent confounding or mediating factors on the relationship between birthweight and adult DNAm. One of these covariates was socioeconomic status as ranked by the postcode-derived Scottish Index of Multiple Deprivation (SIMD). Socioeconomic status is positively associated with birth weight [41], and has been associated with DNAm variability [42]. BMI was included as a covariate in the sensitivity analyses, as is has been shown to associate with birth weight [43], and with DNAm [44]. The number of years of education was also included as educational attainment associates with DNAm in adulthood (most likely via its links to smoking) [45,46], and birth weight has been associated with educational attainment [47]. For detail on acquisition of covariate data see **Supplementary File 1**. Correction for smoking and 20PCs were already included in the EWAS model. Set 1 DNAm data were pre-corrected for batch, cell counts, and relatedness. The fully-adjusted model was:

$$\begin{array}{rl} & CpG{\;}_{\;}birth\;weight\;residuals+age+sex+BMI+SIMD+years\;of\;education\\ & +smoking\left[ever/never\right]+smoking\;pack\;years+20\;methylation\\ & \;PCs+batch\left[set\;2\;only\right]+cell\;counts\left[set2\;only\right] \end{array}$$

### Epigenetic clock analyses

Detail on the estimation of the five epigenetic clocks used have previously been reported elsewhere [22]. Briefly, penalized regression models were used to identify subsets of CpG sites that predict chronological age [23,24], ’phenotypic age’ [25], telomere length [27], and survival [26]. The clocks either trained predictors directly on the outcome (e.g., chronological age or telomere length), or via surrogate markers (e.g., protein levels and biomarkers that are known to associated with biological/health processes). The Horvath clock uses DNAm from 51 tissues and cell types to yield a pan-tissue predictor of chronological age [23]; the Hannum clock also predicts chronological age, but using blood-based DNAm [24]. The more recently developed PhenoAge incorporates blood-based DNAm proxies for clinical biomarkers found to associate with mortality risk, inflammatory and blood-based markers, as well as chronological age, in a ‘phenotypic age’ predictor that associates with healthspan and lifespan [25]. GrimAge takes a similar approach, using blood-based DNAm estimates of seven plasma proteins, as well as smoking pack years and chronological age to predict time to death [26]. Finally, DNAmTL is a blood-based DNAm estimator of telomere length (a measure of cellular ageing), found to outperform measured telomere length in prediction of mortality [27]. The residuals from regressions of these predictors on chronological age gives an index of biological age acceleration. Here, we used linear regression to examine the association between birth weight (outcome) and the five sets of age acceleration residuals, with covariate adjustment for age, sex, methylation ‘set’, and estimated white cell proportions. Correction for multiple testing was carried out using false discovery rate p < 0.05 for epigenetic clock analyses.

### Ethics approval and consent to participate

All components of GS received ethical approval from the NHS Tayside Committee on Medical Research Ethics (REC Reference Number: 05/S1401/89). GS has also been granted Research Tissue Bank status by the Tayside Committee on Medical Research Ethics (REC Reference Number: 10/S1402/20), providing generic ethical approval for a wide range of uses within medical research.

## Results

### Population characteristics

There were 1,757 Generation Scotland (GS) participants with both birth weight and DNAm information available (Table 1). The set 1 population was 59.2% female, with a mean birth weight of 3,377 g (SD = 518 g), and gestational age of 40 weeks (SD = 1.8). The set 2 population was 55.8% female, with a mean birth weight of 3,421 g (SD = 535.4 g), and mean gestational age of 39.7 weeks (SD = 1.5). The minimum birth weight in the sample was 907 g, the maximum was 5,090 g. Using the clinical cut-off of 2,500 g, 4.8% (n = 84) of the sample were of clinically ‘low’ birth weight [14]. This means the data describe DNAm relationships to birth weight across the full spectrum.

**Table 1.** Population characteristics of the Set 1 and Set 2 EWAS samples

	Phenotype Sample			EWAS Sample			Replication Sample		
	**N**	**Mean**	**SD**	**N**	**Mean**	**SD**	**N**	**Mean**	**SD**
Age (years)	4, 710	29.5	10.9	1, 395	37.1	14.7	362	25.8	5.2
Birthweight (g)	4, 710	3, 399	516	1, 395	3, 377	518	362	3, 421	535.4
Gestation (weeks)	4, 710	39.8	1.7	1, 395	40	1.8	362	39.7	1.5
BMI (kg/m2)	4, 385	25.2	5.2	1, 387	26.1	5.4	360	24.9	4.8
Education*	4, 411	5	4–6	1, 317	5	4–6	350	5	4–6
	**N**	**%**		**N**	**%**		**N**	**%**	
Sex – Male	2, 025	43		569	40.8		160	44.2	
Female	2, 688	57		826	59.2		202	55.8	
Socieconomic Status**	*(4, 389)*			*(1, 310)*			*(337)*		
Quintile 1 (most deprived)	621	14.2		207	15.8		65	19.3	
Quintile 2	717	16.3		210	16		57	16.9	
Quintile 3	715	16.3		183	14		62	18.4	
Quintile 4	1, 064	24.2		301	23		72	21.4	
Quintile 5 (least deprived)	1, 272	29		409	31.2		81	24	
Smoking	*(4, 525)*			*(1, 344)*			*(357)*		
Current Smoker	918	20.3		257	19.1		88	24.6	
Ex-Smoker (<12 months)	237	5.2		56	4.2		26	7.3	
Ex-Smoker (>12 months)	662	14.6		273	20.3		48	13.4	
Never Smoker	2, 708	59.8		758	56.4		195	54.6	

* Median and Interquartile range reported. Education was coded as an ordinal variable: 0 = 0 yrs, 1 = 1–4 yrs, 2 = 5–9 yrs, 3 = 10–11 yrs, 4 = 12–13 yrs, 5 = 14–15 yrs, 6 = 16–17 yrs, 7 = 18–19 yrs, 8 = 20–21 yrs, 9 = 22–23 yrs, 10 = ≥24 yrs.

**SIMD Quintile. SIMD is the Scottish Index of Multiple Deprivation, a postcode-derived index of socioeconomic status. The quintiles derived on the full Generation Scotland cohort ranged from 1 (most deprived) to 5 (least deprived).

### Epigenome-wide association study of birthweight

**Figure 2.**
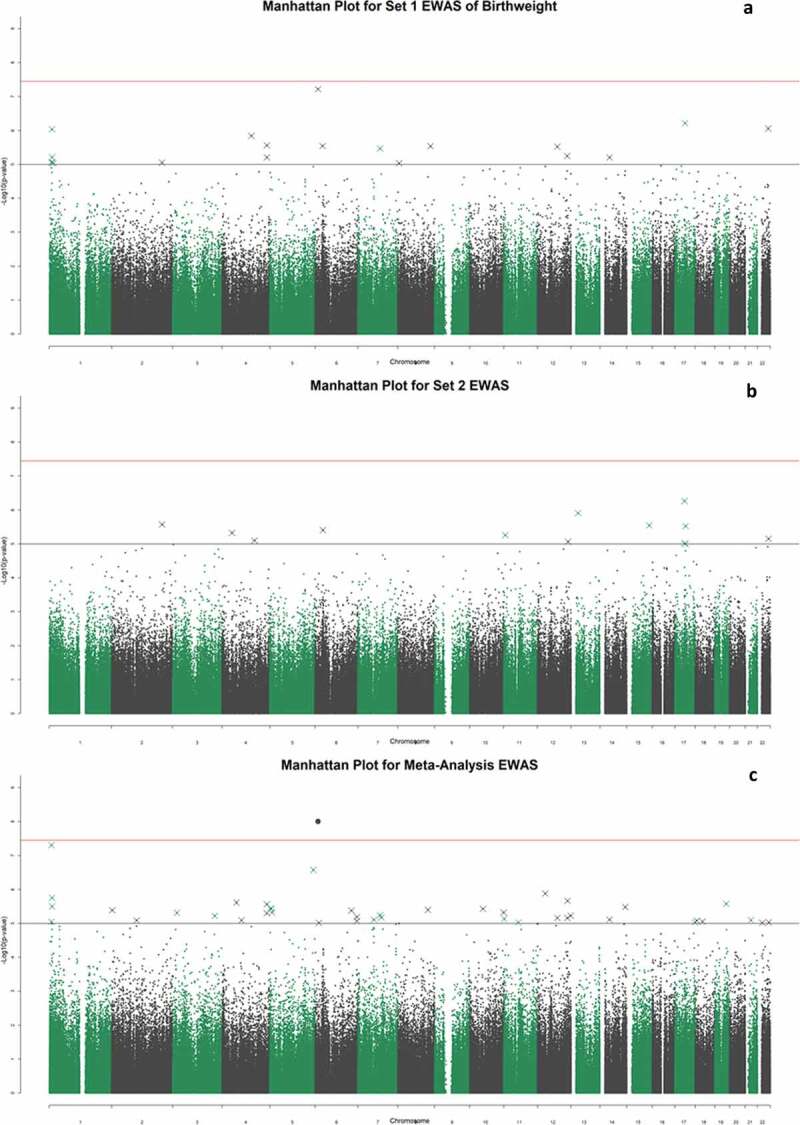
Manhattan plots for the set 1 epigenome-wide association study of birth weight (a); the set 2 sample EWAS (b); and the meta-analysis EWAS (c). The black and red lines represent the suggestive, and genome-wide significant p-value thresholds of P = 1x10^−5^ and 3.6x10^−8^, respectively

The set 1 EWAS of birth weight revealed no CpGs significant at the genome-wide level (p < 3.6x10^−8^ [39];), although 19 CpGs had P < 1x10^−5^ (minimum p-value of 6.05 × 10^−8^ for cg00966482) (Figure 2(a); Supplementary Table 1). The 19 CpGs were largely uncorrelated, with the exception of three CpGs (located within *CASZ1* – two within 200 base pairs of each other, with the third site around 11kb away – Supplementary Table 1) that had absolute r ≥ 0.6.

**Figure 3.**
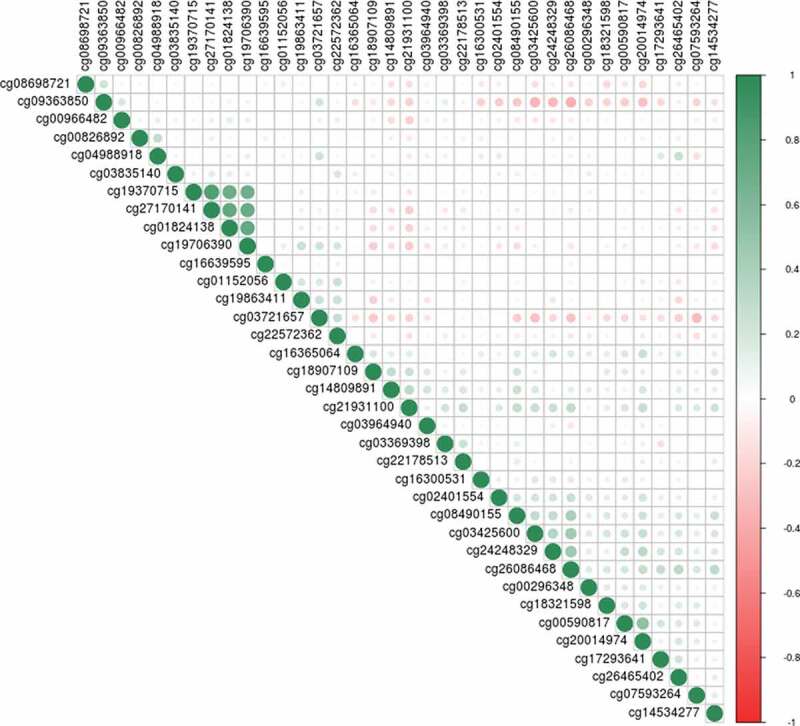
Effect sizes for 18 of the top 19 CpG sites in the set 1 sample plotted against the effect sizes in the set 2 sample (cg04988918 did not pass quality control in the set 2 array). The point size is determined by the -log10 of the p-values for these hits in the set 2 analysis. The two points labelled in black are the two CpG sites which achieved nominal significance in the set 2 study, and the three highlighted in red are the three co-methylated CpG sites within the *CASZ1* gene

There were no genome-wide significant associations in the set 2 sample (n = 362) (Figure 2(b); Supplementary Table 2). Of the 19 CpGs that exceeded p < 1x10^−5^ significance threshold in the set 1 EWAS, two reached nominal significance (p < 0.05) in the set 2 analysis: cg00590817 (p = 0.0069), and cg00966482 (p = 0.031). The latter CpG was the most significantly associated site from the set 1 EWAS. There was moderate concordance between the effect sizes of the top 19 DNAm associations with birthweight in set 1, and the same CpGs in the set 2 analysis (r = 0.59) (Figure 3).

### Meta-analysis EWAS

**Figure 4.**
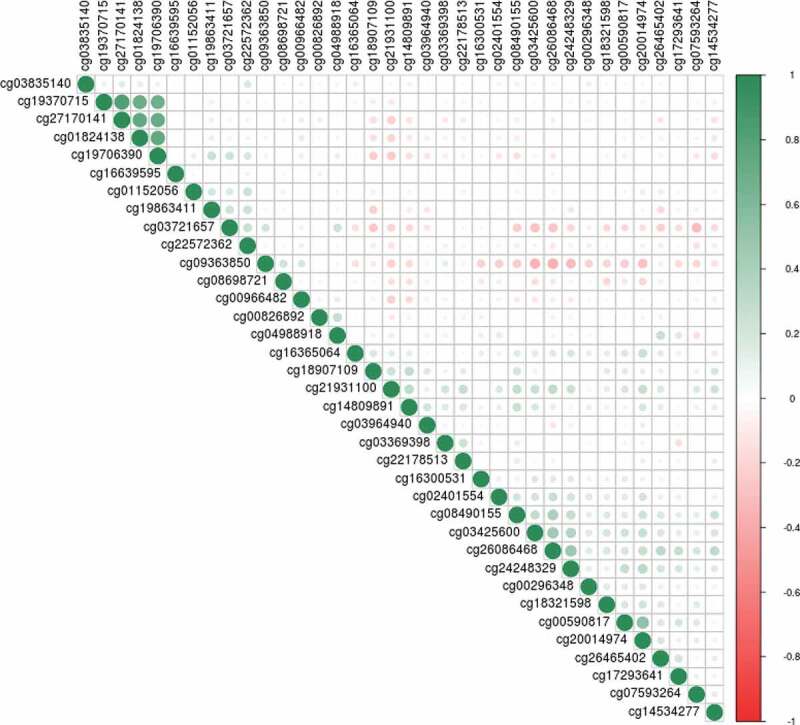
Correlation plot between methylation at the top 36 CpG sites from the meta-analysis EWAS. The shade and scale of the dots represent the magnitude and direction of the correlation between pairs of CpGs

Meta-analysis of the set 1 and set 2 EWAS samples resulted in a genome-wide significant association between birthweight and DNAm at the CpG site cg00966482 mapping to the *HERV-FRD* and *LOC221710/SMIM13* genes (β = 0.0206, SE = 0.0035, p = 5.97x10^−9^; Figure 2(c); Supplementary Table 3). There were 36 CpG sites with p < 1x10^−5^, including four CpGs located within the gene *CASZ1*. These four sites in *CASZ1* were co-methylated, absolute r ≥ 0.92 (Figure 4)

### Subsample analysis excluding preterm births

To address the view that preterm births may represent a distinct group, we ran the EWASs on a subgroup (n_Set1_ = 1,346, n_Set2_ = 351) excluding those with less than 37 complete weeks gestational age at birth (see Supplementary Table 4 for population characteristics). In the meta-analysis EWAS performed in this subgroup, cg00966482 remained significant, with a slightly greater effect (β = 0.0213; SE = 0.0037; p = 5.68x10^−9^). There were 36 CpG sites with p < 1x10^−5^, 23 of which appeared in the list of 36 lead sites in the main meta-analysis EWAS (Supplementary Table 5).

### Adjusting the EWAS model for lifestyle factors

**Figure 5.**
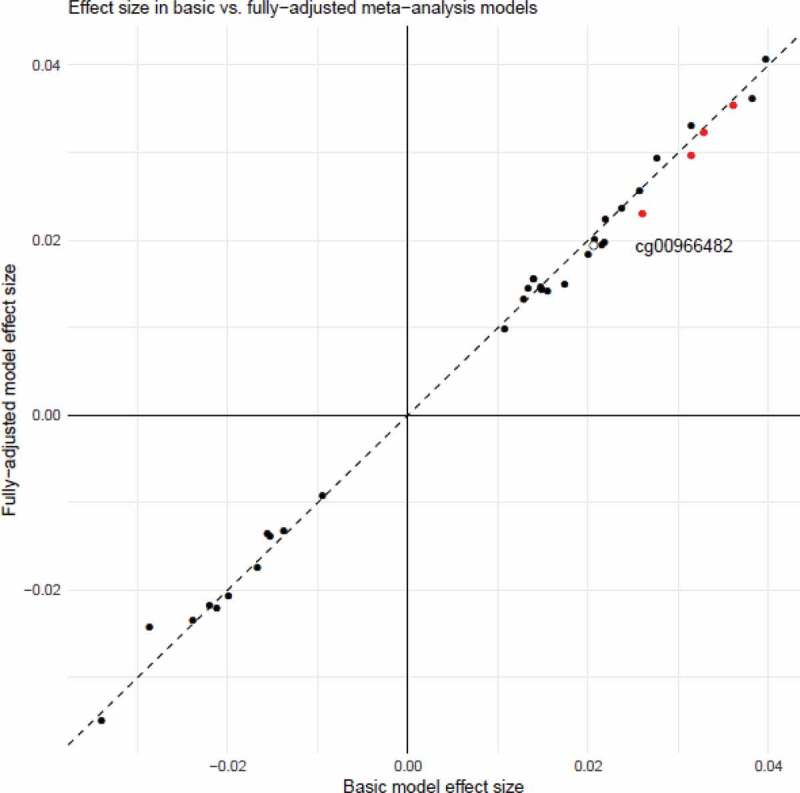
Effect sizes for the main meta-analysis EWAS, and the fully-adjusted sensitivity analysis model. The hollow point labelled in black is cg00966482, in the *HERV-FRD/SMIM13* gene which achieved epigenome-wide significance in the main model meta-analysis. The points in red are in the four CpG sites in the gene *CASZ1* which had p < 1x10^−5^ in the meta-analysis EWAS

As a sensitivity analysis, we re-ran the EWAS pipeline, with corrections for BMI, education, and SIMD separately, and all three covariates together. This fully-adjusted model resulted in some changes to the effect sizes for the 36 sites with p < 1x10^−5^ in the basic meta-analysis output (mean attenuation of 2.2%, range 12.2% attenuation to 15.3% increase in effect size; Supplementary Table 6). There was, however, good concordance between the effect sizes for the top 36 CpG sites identified in the main model, and the same sites in this fully-adjusted sensitivity model (r = 0.998; Figure 5). Full summary statistics for the sensitivity analyses are available at https://doi.org/10.7488/ds/2876.

### Relationship of birth weight to epigenetic signatures of age and telomere length

The mean values for the five epigenetic clocks were: Horvath age 44.7 yrs (SD = 10.6); Hannum age 36.1 yrs (SD = 10.2); PhenoAge 32.8 yrs (SD = 12); GrimAge 45.7 yrs (SD = 14.1); DNAm telomere length 7.6 kilobase pairs (SD = 0.29). Linear regression models of birth weight residuals (outcome) against five signatures of epigenetic age acceleration (the scaled residuals from regression of the clock predictors on chronological age) revealed significant associations between higher birth weight and lower Grim Age acceleration (β = −0.083; SE = 0.026, p_(FDR)_ = 3.6x10^−3^) and longer DNAm telomere length (β = 0.098; SE = 0.027, p_(FDR)_ = 1.7x10^−3^; Table 2). Associations between birth weight and the other three measures of epigenetic age acceleration were non-significant (Table 2).

**Table 2.** Outputs of linear regression models between birth weight residuals and five epigenetic signatures of accelerated biological ageing – Horvath (Intrinsic Epigenetic Age Acceleration; IEAA), Hannum, PhenoAge, GrimAge, and DNAmTL

Clock	Standardised Beta	SE	P	P.adj
Horvath	3.2x10^−3^	0.025	0.90	0.90
Hannum	−6.2x10^−3^	0.027	0.82	0.90
GrimAge	−0.083	0.026	1.4x10^−3^	3.6x10^−3^
PhenoAge	−0.042	0.027	0.12	0.20
DNAmTL	0.098	0.027	3.3x10^−4^	1.7x10^−3^

## Discussion

We identified one epigenome-wide significant association between birth weight and blood-based DNA methylation in adulthood, and significant associations between birth weight and two epigenetic age measures.

In the EWAS meta-analysis we observed a genome-wide significant association between higher birthweight and higher methylation levels at cg00966482 (mapping to *HERV-FRD* and *LOC221710/SMIM13*). *HERV-FRD* encodes syncitin-2, a protein involved in placental embedding, whereas *LOC221710/SMIM13* is a gene with unknown functions [48]. This site was not identified in a previous EWAS meta-analysis of birthweight in newborns [18]. Of the 36 CpG sites with P < 1x10^−5^ in the meta-analysed EWAS, 20 were located within known genes. Eighteen of the 20 sites were within regions with evidence for transcription factor binding, open chromatin, or DNase hypersensitivity. These may be attractive candidates for further investigation of the relationship between birth weight, DNA methylation, and transcriptional activity (Supplementary Table 3). Several of these genes contain SNPs that have genome-wide significant associations (GWAS P < 5x10^−8^) with cardiovascular, psychiatric, and developmental pathways (Supplementary Table 7). Four of the 36 CpG sites exceeding p < 1x10^−5^ identified in the meta-analysis EWAS were highly correlated (min r = 0.92, Figure 4). Higher birthweight was associated with higher methylation levels at these sites, which were located within *CASZ1*, a gene encoding the zinc finger protein castor homolog 1, a transcriptional activator involved in vascular morphogenesis [49]. A differentially methylated region in the *CASZ1* gene was recently identified in placental tissue between infants born small vs. large for gestational age [17]. Additionally, a recent epigenome-wide meta-analysis of gestational age in children and infants reports several significant sites within *CASZ1* [50]. In these studies *CASZ1* methylation was positively associated with both size and gestational age. The findings presented here, although non-significant (p ≥ 4.37x10^−7^), suggest that future studies might wish to focus on studying this relationship between birth weight and *CASZ1* methylation in further detail. Genetic variants in *CASZ1* have previously been implicated by GWAS in various aspects of cardiovascular health (Supplementary Table 7). These have included studies in multi-ethnic populations on blood pressure [51,52], and on other cardiovascular health issues such as atrial fibrillation [53] and stroke [54].

Sensitivity analyses that excluded preterm births or adjusted individually and collectively for a series of possible confounder/mediator lifestyle variables resulted in only minor changes to the effect sizes of the primary model. However, the covariate adjustments did result in increased p-values for all CpG sites, suggesting that replication in larger samples and further work to investigate the possibility of mediation by lifestyle factors should be considered. The analysis excluding preterm births identified the same epigenome-wide significant site (cg00966482, *HERV-FRD/SMIM13* locus) as the primary analysis. However, there was an increase in the effect size, resulting in a lower p-value for this CpG (p = 5.68x10^−9^). There were some differences between the CpGs identified at a suggestive epigenome-wide threshold of p < 1x10^−5^ (36 in both analyses). This may indicate differences in the biological pathways associated with birth weight in adults, according to the length of gestation, and would be an interesting topic for further study.

Significant associations are demonstrated between birth weight and epigenetic predictors of ageing, mortality, and cellular senescence. Higher birth weight is associated with lower GrimAge acceleration [26] and longer telomeres as estimated by DNAm data [27]. The effect sizes are relatively small; for instance, with astandardised Beta value of −0.083 between GrimAge acceleration and birth weight. These results, however modest, are supportive of the known associations between low birth weight and a broad range of adverse health outcomes. We did not find significant associations between birth weight and Horvath Age [23], Hannum Age [24], or PhenoAge [25] here. We have previously found that GrimAge outperforms the other clocks in the prediction of self-reported disease burden and clinical traits in GS [22], and the creators of GrimAge reported its superiority in predicting onset of a host of health conditions [26]. The DNAm estimate of telomere length performs a different function than the other epigenetic age estimators, as it specifically predicts telomere length – a measure of cellular age. The DNAmTL result suggests a relationship between lower birth weight and accelerated ageing at a cellular level, while the GrimAge result demonstrates this at a whole-body level. It is of interest that an association exists between birth weight and some, but not all, of the epigenetic age predictors tested. It is possible that some of the specific variables included in the different clocks might underlie this observation. For instance, GrimAge contains a methylation-based proxy for leptin [26], a hormone involved in regulating food intake and energy expenditure, which is likely to be linked to adult weight. In turn, adult weight associates with birth weight [43]. In contrast, PhenoAge is calculated based on methylation proxies primarily of inflammatory processes, which may not associate with birth weight as strongly; meanwhile the Hannum and Horvath clocks are more broadly trained on age, and so may not be picking up the same level of detail on susceptibility to ill-health. Maternal GrimAge acceleration during pregnancy has recently been associated with shorter gestation and, independently, lower birthweight of the offspring in a small sample of pregnant women [55]. This indicates the potential utility of GrimAge in describing both processes relevant to foetal development, and the later-life consequences of the foetal experience.

### Strengths and limitations

This study exploited rarely available data linkage capacity to acquire neonatal information from birth medical records. This was then linked to DNAm in a large adult sample. There are, however, some limitations inherent to the design of this study. Longitudinal data would allow analysis of the persistence of DNAm signatures across time, although this has been investigated in previous studies [18]. A further limitation to this study is the lack of maternal characteristics available for inclusion in our models. As GS is a cross-sectional study, and birth information was derived from multiple historical sources, this information was not consistently available for our sample.

DNAm data were derived from whole blood taken during adulthood, so our findings might not generalize to other tissue types. However, there are advantages to interrogating blood-based methylation levels: first, it is the only tissue type that is readily available in large epidemiological cohorts; and second, it is systemic and tracks multiple biological processes including biomarkers of inflammation, cardiovascular disease, cardiometabolic disease, all of which are relevant processes related to birthweight.

## Conclusions

This study presents the first epigenome wide association study of birth weight on DNA methylation in adulthood, alongside associations between birth weight and both epigenetic age acceleration and telomere length. The Developmental Origins of Health and Disease theory predicts that birth weight, which may be seen as a marker of the intrauterine environment, is associated with outcomes in many domains of adult health, and it has been proposed that these associations may be reflected in the epigenome. We found evidence to support this at both the level of single CpG site methylation, and at the level of broader mortality and health-related DNAm phenotypes.

## Supplementary Material

Supplemental Material

## Data Availability

According to the terms of consent for Generation Scotland participants, access to data must be reviewed by the Generation Scotland Access Committee. Applications should be made to access@generationscotland.org. Summary statistics for the EWAS models will be made available on the University of Edinburgh DataShare facility upon acceptance **https://doi.org/10.7488/ds/2876.**
