# Supplementary material for: Birth weight associations with DNA methylation differences in an adult population
Source: Epigenetics. 2020 Oct 20;16(7):783–96. doi: 10.1080/15592294.2020.1827713 (PMC8216207; doi:10.1080/15592294.2020.1827713)
Supplement: Supplemental Material [file KEPI_A_1827713_SM1698.docx]

**Supplementary File 1:** Identification and calculation of variables in Generation Scotland.

Identifying birth weight information:

Birth weight in grams, alongside Gestational Age and twin information was merged into Generation Scotland, using a linker file containing the respective identifiers for each cohort, and any matching GS IDs.

In the Walker record, some birth weight information was stored only in pounds and ounces. For these data points, the following calculation was used to impute birthweight in grams:

(no.lbs/453.59) + (no.oz/28.35) = birth weight in grams

In SMR02, data was stored under the identifier of the mother, rather than the child. The child’s sex, date of birth, and the mother’s GS ID were used to create a ‘key’ in the format “Maternal ID*sex*DOB”, making a unique identifier for each individual allowing for the record to be merged.

Different-sex twin pairs in SMR02 were separated by birth order, and merged with GS in the same manner as singleton births. Same sex twin pairs were excluded if the birth weight discordance exceeded 1SD of the group birth weights. For those remaining, one of each twin pair was randomly excluded – therefore for all same sex twins, one twin’s birth weight info was linked to one adult Generation Scotland record. In SMR 11, a similar process was undertaken, as some same-sex twin pairs were recorded under identical ID numbers. Again, one twin from each pair was randomly excluded. 19 twin pairs in SMR11, and 24 in SMR02 had their data imputed in this way.

Collection of Physical and Biochemical Measures in GS [1]:

Measurements and samples were taken at a clinic visit by probands. This included blood sampling from which DNA methylation was analysed. Height (cm) and weight (kg) were also measured, from which body mass index (BMI) was calculated (weight/height^2^).

Other lifestyle information collected in GS:

The Scottish Index of Multiple Deprivations (SIMD) is a postcode-derived measure of socioeconomic status (SES) local to Scotland. This was ascertained based on probands’ current postcodes, and was available both as a ranking and divided into quintiles (1= deprived : 5= least deprived). A Pre-Clinical Questionnaire (PCQ) was administered to GS participants before their clinic visit. This was a general health questionnaire asking about physical conditions and symptoms, and lifestyle factors such as diet and smoking. The smoking section included self-report of lifetime smoking status (currently smoke/stopped within 12 months/stopped more than 12 months ago/never smoked), as well as information on how heavily the proband smoked and for how long.

See <https://www.ed.ac.uk/generation-scotland/our-resources/scottish-family-health-study> for a complete version of the questionnaire.

References:

1. Smith, B.H., et al., *Cohort Profile: Generation Scotland: Scottish Family Health Study (GS:SFHS). The study, its participants and their potential for genetic research on health and illness.* International Journal of Epidemiology, 2013. **42**(3): p. 689-700.

**Supplementary File 2:** Details of DNA methylation data acquisition and quality control.

Data came from the family-based Generation Scotland: Scottish Family Health Study (GS). GS participants were recruited from GP practices in five regions across Scotland between the years 2006 and 2011 [1]. The probands were aged between 35 and 65 years and were asked to invite first degree relatives to join the study, which had a final sample size of 23, 690. A variety of cognitive, physical, and health data were collected at the study baseline along with blood samples for DNA genotyping.

Blood-based DNA methylation data were obtained on a subset of 5, 200 participants using the Illumina EPIC array. Quality control details have been reported previously Briefly, probes were removed based on: (i) outliers from visual inspection of the log median intensity of the methylated versus unmethylated signal per array; (ii) a beadcount <3 in more than 5% samples and; (iii) ≥5% of samples having a detection p-value >0.05; (iii) any non-autosomal or non-CpG sites, cross-hybridising probes or sites with a SNP at the target CpG or site of single base extension [2]. Samples were removed (i) if there was a mismatch between their predicted sex and recorded sex and/or (ii) if ≥1% of CpGs had a detection p-value >0.05 [3]. We also excluded 3 individuals who answered ‘yes’ to all self-reported disease questions.

As reported in Bermingham et al [3]: “The M-values for CpGs on autosomal chromosomes were pre-corrected for relatedness, estimated blood cell types and processing batch using DISSECT. This was achieved by saving the residuals from a mixed linear model that included methylation as the dependent variable and the following predictor variables: a genetic relatedness matrix fitted in a leave-one-chromosome-out fashion (i.e. SNPs on the same chromosome as the CpG were excluded); proportions of granulocytes, natural killer cells, B-cells, CD4+ T-cells and CD8+ T-cells estimated using the estimateCellCounts function in minfi; and a variable that indicated the batch in which array hybridisation, staining and scanning took place.”

Multi-dimensional scaling was used [4] to generate epigenetic principal co-ordinates on the methylation data after regressing out age, sex, and processing batch. The first 20 PCs were included as covariates to account for additional heterogeneity [3].

For the present analyses, we considered individuals from the DNA methylation subset of GS with birthweight information available, called ‘set 1’. The analysed dataset comprised 841, 753 probes and 1, 395 samples.

For the set 2 dataset, Illumina HumanMethylationEPIC BeadChips were used to profile genome-wide DNA methylation in whole blood samples from 4, 683 unrelated (<0.05) GS participants. These participants were also unrelated (<0.05) to those in the set 1 dataset. Quality control of the raw intensity data was carried out using meffil [5] and shinyMethyl [6]. The .idat files were read into R [7] using either the meffil.qc function for the meffil pipeline or minfi’s read.metharray function for shinyMethyl. Quality control was initially carried out using meffil, which was used to remove samples (i) for which a discrepancy between self-reported and methylation-predicted sex (based on the difference between the median copy number intensity for the Y chromosome and the median copy number intensity for the X chromosome) was identified; (ii) that had > 1% CpGs with a detection *p*-value > 0.05; (iii) that showed evidence of dye bias; (iv) that were outliers for the bisulphite conversion control probes; or (v) that had a median methylated signal intensity more than three standard deviations lower than expected. Following the removal of the poor-performing samples detected by meffil, shinyMethyl was used to perform a second round of quality control, as described for the set 1 methylation dataset. Following the exclusion of samples identified by the steps above, MDS plots were inspected for additional sample outliers and these were excluded too. Poor-performing probes were then identified and removed using meffil. Probes were deemed to have failed if (i) they had a beadcount of < 3 in > 5% samples or (ii) more than > 5% samples had a detection *p*-value > 0.05.

References:

1. Smith, B.H., et al., *Cohort Profile: Generation Scotland: Scottish Family Health Study (GS:SFHS). The study, its participants and their potential for genetic research on health and illness.* International Journal of Epidemiology, 2013. **42**(3): p. 689-700.

2. McCartney, D.L., et al., *Identification of polymorphic and off-target probe binding sites on the Illumina Infinium MethylationEPIC BeadChip.* Genomics Data, 2016. **9**: p. 22-24.

3. Bermingham, M.L., et al., *Identification of novel differentially methylated sites with potential as clinical predictors of impaired respiratory function and COPD.* bioRxiv, 2018: p. 473629.

4. Lê, S., J. Josse, and F. Husson, *FactoMineR: an R package for multivariate analysis.* Journal of statistical software, 2008. **25**(1): p. 1-18.

5. Min, J., et al., *Meffil: efficient normalization and analysis of very large DNA methylation datasets.* Bioinformatics, 2018. **34**(23): p. 3983-3989.

6. Fortin, J.P., E. Fertig, and K. Hansen, *shinyMethyl: interactive quality control of Illumina 450k DNA methylation arrays in R.* F1000Res, 2014. **3**: p. 175.

7. Team, R.C., *R: A language and environment for statistical computing*. 2018, R Foundation for Statistical Computing: Vienna, Austria.

**Supplementary Table 1:** The 19 CpG sites with P< 1x10^-5^ for their association with birth weight in the Set 1 EWAS (n=1,395).

| **Probe ID** | **Gene** | **HG19.coordinates** | **Beta** | **Standard Error** | **P Value** |
| --- | --- | --- | --- | --- | --- |
| cg00966482 | *HERV-FRD/SMIM13* | chr6:11111926 | 0.020 | 0.004 | 6.05x10^-8^ |
| cg06708560 | *DNAJC7/NKIRAS2* | chr17:40170084 | 0.023 | 0.005 | 6.11x10^-7^ |
| cg07593264 | | chr22:43410455 | -0.018 | 0.004 | 8.75x10^-7^ |
| cg27170141 | *CASZ1* | chr1:10711044 | 0.032 | 0.007 | 9.22x10^-7^ |
| cg21931100 | | chr4:116633269 | -0.023 | 0.005 | 1.44x10^-6^ |
| cg00826892 | | chr4:178366013 | 0.032 | 0.007 | 2.75x10^-6^ |
| cg16908601 | *OR2B3* | chr6:29055885 | -0.031 | 0.007 | 2.86x10^-6^ |
| cg15090897 | | chr8:130286226 | -0.022 | 0.005 | 2.91x10^-6^ |
| cg03369398 | | chr12:78973332 | -0.026 | 0.006 | 2.99x10^-6^ |
| cg16639595 | *SRI* | chr7:87856984 | 0.015 | 0.003 | 3.39x10^-6^ |
| cg16300531 | *KSR2* | chr12:118405988 | 0.040 | 0.009 | 5.65x10^-6^ |
| cg19370715 | *CASZ1* | chr1:10710847 | 0.026 | 0.006 | 6.15x10^-6^ |
| cg04988918 | | chr4:178366394 | 0.013 | 0.003 | 6.22x10^-6^ |
| cg08490155 | *SSTR1* | chr14:38676796 | -0.017 | 0.004 | 6.27x10^-6^ |
| cg00590817 | | chr1:8272081 | 0.026 | 0.006 | 8.62x10^-6^ |
| cg01824138 | *CASZ1* | chr1:10699604 | 0.034 | 0.008 | 8.76x10^-6^ |
| cg13524161 | | chr2:200715930 | -0.022 | 0.005 | 8.86x10^-6^ |
| cg16004427 | | chr1:16083101 | 0.028 | 0.006 | 9.19x10^-6^ |
| cg23154832 | *CSMD1* | chr8:4583355 | -0.028 | 0.006 | 9.19x10^-6^ |

**Supplementary Table 2:** CpGs with p<1x10^‑5^ in the Set 2 EWAS (n=362).

| **Probe ID** | **Gene** | **HG19.coordinates** | **Beta** | **Standard Error** | **P Value** |
| --- | --- | --- | --- | --- | --- |
| cg04308185 | *ORMDL3* | chr17:38084377 | 0.0811 | 0.016 | 5.4x10^-7^ |
| cg11354629 | *GSX1* | chr13:28366598 | -0.0619 | 0.013 | 1.2x10^-6^ |
| cg19641384 |  | chr2:201694128 | -0.0672 | 0.014 | 2.7x10^-6^ |
| cg19567891 | *LOC254559* | chr15:89921083 | 0.0768 | 0.016 | 2.9x10^-6^ |
| cg22078805 | *FAM171A2* | chr17:42432046 | 0.112 | 0.024 | 3.0x10^-6^ |
| cg02247838 | *CCHCR1* | chr6:31110639 | 0.0402 | 0.009 | 4.0x10^-6^ |
| cg00780250 | *PDS5A* | chr4:39979372 | 0.101 | 0.022 | 4.7x10^-6^ |
| cg24066259 | *CCKBR* | chr11:6281383 | 0.128 | 0.028 | 5.5x10^-6^ |
| cg01503807 | *PARVB* | chr22:44560047 | -0.0525 | 0.011 | 7.0x10^-6^ |
| cg13538398 |  | chr4:129718449 | 0.0384 | 0.008 | 8.0x10^-6^ |
| cg00792008 | *TMEM120B* | chr12:122189621 | 0.0409 | 0.009 | 8.6x10^-6^ |
| cg15942003 | *ORMDL3* | chr17:38084581 | 0.0546 | 0.012 | 9.7x10^-6^ |
| cg23766254 | *FAM171A2* | chr17:42431859 | 0.111 | 0.025 | 9.98x10^-6^ |

**Supplementary Table 3:** CpGs with p<1x10^‑5^ in the meta-analysis of set 1 EWAS and set 2 EWAS, with Beta, Standard Error and P-values for Set 1, Set 2, and meta-analysis EWASs. Cg04988918 did not pass quality control in the set 2 sample array. CpG sites in bold denote sites with p<1x10^‑5^ in the set 1 EWAS.

|  |  |  |  | **Set 1 (n=1,395)** | | | **Set 2 (n=362)** | | | **Meta-analysis (n=1,757)** | | |  |
| --- | --- | --- | --- | --- | --- | --- | --- | --- | --- | --- | --- | --- | --- |
| **Probe ID** | **Gene** | **Functional Annotation** | **HG19.coordinates** | **Beta** | **SE** | **P Value** | **Beta** | **SE** | **P Value** | **Beta** | **SE** | **P Value** | **Direction of DNAm across samples** |
| **cg00966482** | *HERV-FRD /SMIM13* | DHS | chr6:11111926 | 0.020 | 0.004 | 6.05 x10^-8^ | 0.030 | 0.014 | 0.031 | 0.021 | 0.004 | 5.97x10^-9^ | ++ |
| **cg00590817** |  | DHS | chr1:8272081 | 0.026 | 0.006 | 8.62 x10^-6^ | 0.042 | 0.015 | 0.007 | 0.028 | 0.005 | 2.84x10^-7^ | ++ |
| cg16365064 |  | DHS | chr5:172984486 | 0.020 | 0.005 | 1.16 x10^-5^ | 0.024 | 0.009 | 0.009 | 0.021 | 0.004 | 3.24x10^-7^ | ++ |
| **cg27170141** | *CASZ1* | Open Chromatin | chr1:10711044 | 0.032 | 0.007 | 9.22 x10^-7^ | 0.024 | 0.020 | 0.241 | 0.031 | 0.006 | 4.37x10^-7^ | ++ |
| cg18321598 |  | DHS | chr12:30684738 | 0.017 | 0.004 | 1.13 x10^-5^ | 0.018 | 0.009 | 0.035 | 0.017 | 0.004 | 1.03x10^-6^ | ++ |
| **cg00826892** |  | TFBS | chr4:178366013 | 0.032 | 0.007 | 2.75 x10^-6^ | 0.024 | 0.021 | 0.252 | 0.031 | 0.007 | 1.34x10^-6^ | ++ |
| cg26465402 | *ADCY2* | TFBS | chr5:7803274 | 0.010 | 0.003 | 5.60 x10^-5^ | 0.013 | 0.005 | 0.007 | 0.011 | 0.002 | 1.37x10^-6^ | ++ |
| **cg19370715** | *CASZ1* | Open Chromatin | chr1:10710847 | 0.026 | 0.006 | 6.15 x10^-6^ | 0.025 | 0.017 | 0.137 | 0.026 | 0.005 | 1.78x10^-6^ | ++ |
| cg02401554 |  |  | chr4:57203385 | 0.012 | 0.003 | 1.95 x10^-5^ | 0.014 | 0.007 | 0.036 | 0.013 | 0.003 | 1.87x10^-6^ | ++ |
| cg08698721 | *MEG3* | DHS | chr14:101294147 | -0.015 | 0.003 | 1.57 x10^-5^ | -0.018 | 0.009 | 0.056 | -0.015 | 0.003 | 2.24x10^-6^ | -- |
| **cg16639595** | *SRI* |  | chr7:87856984 | 0.015 | 0.003 | 3.39 x10^-6^ | 0.033 | 0.038 | 0.382 | 0.015 | 0.003 | 2.34x10^-6^ | ++ |
| cg22178513 | *A1CF* |  | chr10:52583805 | -0.034 | 0.008 | 1.50 x10^-5^ | -0.036 | 0.021 | 0.081 | -0.034 | 0.007 | 2.86x10^-6^ | -- |
| **cg16300531** | *KSR2* | TFBS; Open Chromatin; | chr12:118405988 | 0.040 | 0.009 | 5.65 x10^-6^ | 0.032 | 0.030 | 0.292 | 0.04 | 0.009 | 3.02x10^-6^ | ++ |
| **cg03369398** |  |  | chr12:78973332 | -0.026 | 0.006 | 2.99 x10^-6^ | -0.011 | 0.014 | 0.441 | -0.024 | 0.005 | 3.38x10^-6^ | -- |
| cg17293641 | *TNFRSF11B* | DHS | chr8:119964144 | 0.020 | 0.005 | 2.31 x10^-5^ | 0.021 | 0.011 | 0.060 | 0.02 | 0.004 | 3.41x10^-6^ | ++ |
| cg03425600 |  | DHS | chr12:132648585 | 0.014 | 0.003 | 1.88 x10^-5^ | 0.012 | 0.007 | 0.079 | 0.014 | 0.003 | 3.76x10^-6^ | ++ |
| cg22572362 | *SLC9A3* | DHS | chr5:501938 | 0.025 | 0.006 | 1.73 x10^-5^ | 0.027 | 0.016 | 0.098 | 0.026 | 0.006 | 3.84x10^-6^ | ++ |
| cg14809891 |  | Open Chromatin | chr5:5739504 | -0.021 | 0.005 | 2.40 x10^-5^ | -0.023 | 0.013 | 0.071 | -0.021 | 0.005 | 4.10x10^-6^ | -- |
| **cg08490155** | *SSTR1* | DHS | chr14:38676796 | -0.017 | 0.004 | 6.27 x10^-6^ | -0.010 | 0.011 | 0.362 | -0.017 | 0.004 | 4.56x10^-6^ | -- |
| **cg01824138** | *CASZ1* | DHS | chr1:10699604 | 0.034 | 0.008 | 8.76 x10^-6^ | 0.024 | 0.021 | 0.261 | 0.032 | 0.007 | 4.65x10^-6^ | ++ |
| cg14534277 | *DSC3* | DHS | chr18:28623874 | -0.019 | 0.005 | 2.48 x10^-4^ | -0.037 | 0.012 | 0.003 | -0.022 | 0.005 | 5.04x10^-6^ | -- |
| cg00296348 |  | Open Chromatin | chr10:134721479 | 0.023 | 0.005 | 1.60 x10^-5^ | 0.026 | 0.018 | 0.153 | 0.024 | 0.005 | 5.13x10^-6^ | ++ |
| cg19706390 | *CASZ1* | DHS | chr1:10709702 | 0.036 | 0.008 | 1.71 x10^-5^ | 0.035 | 0.024 | 0.153 | 0.036 | 0.008 | 5.47x10^-6^ | ++ |
| cg18907109 | *HEPACAM2* | TFBS | chr7:92849682 | -0.010 | 0.002 | 1.95 x10^-5^ | -0.008 | 0.005 | 0.124 | -0.01 | 0.002 | 5.57x10^-6^ | -- |
| **cg04988918** |  | DHS | chr4:178366394 | 0.013 | 0.003 | 6.22 x10^-6^ | *NA* | *NA* | *NA* | 0.013 | 0.003 | 5.72x10^-6^ | +? |
| **cg21931100** |  | TFBS | chr4:116633269 | -0.023 | 0.005 | 1.44 x10^-6^ | -0.001 | 0.012 | 0.917 | -0.02 | 0.004 | 6.35x10^-6^ | -- |
| cg24248329 | *NFYC* | TFBS; Open Chromatin | chr1:41175132 | 0.021 | 0.005 | 4.63 x10^-5^ | 0.027 | 0.014 | 0.055 | 0.022 | 0.005 | 6.82x10^-6^ | ++ |
| cg03835140 | *NKPD1* | TFBS; Open Chromatin | chr19:45662154 | 0.013 | 0.003 | 1.26x10^-4^ | 0.036 | 0.012 | 0.004 | 0.015 | 0.003 | 7.08x10^-6^ | ++ |
| cg03964940 | *STX11* | DHS | chr6:144477779 | 0.021 | 0.005 | 5.52 x10^-5^ | 0.038 | 0.018 | 0.031 | 0.022 | 0.005 | 7.24x10^-6^ | ++ |
| cg01152056 |  | TFBS | chr6:166260319 | 0.035 | 0.009 | 1.07x10^-4^ | 0.062 | 0.026 | 0.015 | 0.038 | 0.009 | 7.54x10^-6^ | ++ |
| **cg07593264** |  | DHS | chr22:43410455 | -0.018 | 0.004 | 8.75 x10^-7^ | 0.007 | 0.011 | 0.536 | -0.016 | 0.004 | 7.60x10^-6^ | -+ |
| cg09363850 |  |  | chr11:58090434 | -0.028 | 0.007 | 1.99 x10^-5^ | -0.039 | 0.030 | 0.189 | -0.029 | 0.006 | 8.03x10^-6^ | -- |
| cg03721657 | *HCCA2* | DHS | chr11:1571033 | 0.014 | 0.004 | 4.32x10^-4^ | 0.024 | 0.008 | 0.004 | 0.016 | 0.004 | 8.65x10^-6^ | ++ |
| cg20014974 |  | DHS | chr1:8271918 | 0.015 | 0.003 | 1.25 x10^-5^ | 0.008 | 0.008 | 0.291 | 0.014 | 0.003 | 8.75x10^-6^ | ++ |
| cg26086468 |  | TFBS; Open Chromatin | chr18:5628160 | -0.012 | 0.003 | 6.72x10^-4^ | -0.022 | 0.007 | 0.002 | -0.014 | 0.003 | 9.90x10^-6^ | -- |
| cg19863411 | *PCYT2* | DHS | chr17:79869801 | 0.022 | 0.006 | 9.93 x10^-5^ | 0.023 | 0.011 | 0.039 | 0.022 | 0.005 | 9.98x10^-6^ | ++ |

**Supplementary Table 4:** Population characteristics of the Set 1 and Set 2 EWAS samples excluding preterm births (<37 weeks gestation).

|  | **Set 1 excl. preterms** | | | **Set 2 excl. preterms** | | |
| --- | --- | --- | --- | --- | --- | --- |
|  | **n** | **Mean** | **SD** | **n** | **Mean** | **SD** |
| Age (years) | 1,346 | 37.1 | 14.6 | 351 | 25.8 | 5.2 |
| Birthweight (g) | 1,346 | 3,419.9 | 459.2 | 351 | 3,449.8 | 500.7 |
| Gestation (weeks) | 1,346 | 40.2 | 1.3 | 351 | 39.9 | 1.2 |
| BMI (kg/m2) | 1,338 | 26.1 | 5.4 | 350 | 24.9 | 4.8 |
| Education* | 1,271 | 5 | 4-6 | 339 | 5 | 4-6 |
|  | **n** | **%** |  | **n** | **%** |  |
| Sex - Male | 548 | 40.7 |  | 153 | 43.6 |  |
| Female | 798 | 59.3 |  | 198 | 56.4 |  |
| Socieconomic Status** | *(1,265)* |  |  | *(326)* |  |  |
| Quintile 1 (most deprived) | 198 | 15.7 |  | 61 | 18.7 |  |
| Quintile 2 | 202 | 15.97 |  | 55 | 16.9 |  |
| Quintile 3 | 180 | 14.2 |  | 60 | 18.4 |  |
| Quintile 4 | 288 | 22.8 |  | 70 | 22.5 |  |
| Quintile 5 (least deprived) | 397 | 31.4 |  | 80 | 24.5 |  |
| Smoking | *(1,297)* |  |  | *(346)* |  |  |
| Current Smoker | 246 | 18.97 |  | 84 | 24.3 |  |
| Ex-Smoker (<12 months) | 53 | 4.1 |  | 25 | 7.2 |  |
| Ex-Smoker (>12 months) | 266 | 20.5 |  | 48 | 13.9 |  |
| Never Smoker | 732 | 56.4 |  | 189 | 54.6 |  |

* Median and Interquartile range reported. Education was coded as an ordinal variable: 0 = 0yrs, 1 = 1-4yrs, 2 = 5-9yrs, 3 = 10-11yrs, 4 = 12-13yrs, 5 = 14-15yrs, 6 = 16-17yrs, 7 = 18-19yrs, 8 = 20-21yrs, 9 = 22-23yrs, 10 = ≥24yrs.

**SIMD Quintile. SIMD is the Scottish Index of Multiple Deprivation, a postcode-derived index of socioeconomic status. The quintiles derived on the full Generation Scotland cohort ranged from 1 (most deprived) to 5 (least deprived).

**Supplementary Table 5:** CpGs with p<1x10^-5^ in the meta-analysed EWAS run on a subgroup of the data excluding preterm births (n=1,697), with Beta, Standard Error, and P-value for Set 1, Set 2, and meta-analysis EWASs. CpG sites in bold denote sites with p<1x10^-5^ in the main meta-analysis EWAS.

|  |  |  | **Set1 excl. preterms (n=1,346)** | | | **Set2 excl. preterms (n=351)** | | | **Meta excl. preterms (n=1,697)** | | |  |
| --- | --- | --- | --- | --- | --- | --- | --- | --- | --- | --- | --- | --- |
| **ProbeID** | **Gene** | **HG19 coordinates** | **Effect** | **SE** | **P** | **Effect** | **SE** | **P** | **Effect** | **SE** | **P** | **Direction of DNAm across samples** |
| **cg00966482** | *HERV-FRD /SMIM13* | chr6:11111926 | 0.0209 | 0.0038 | 3.81 x10^-8^ | 0.0269 | 0.0144 | 0.062 | 0.0213 | 0.0037 | 5.68 x10^-9^ | ++ |
| **cg00590817** |  | chr1:8272081 | 0.0266 | 0.0059 | 7.15 x10^-6^ | 0.0469 | 0.0159 | 0.003 | 0.0291 | 0.0055 | 1.49 x10^-7^ | ++ |
| **cg27170141** | *CASZ1* | chr1:10711044 | 0.0310 | 0.0068 | 5.16 x10^-6^ | 0.0411 | 0.0211 | 0.052 | 0.0319 | 0.0064 | 7.29 x10^-7^ | ++ |
| **cg16365064** |  | chr5:172984486 | 0.0194 | 0.0047 | 3.64 x10^-5^ | 0.0256 | 0.0097 | 0.008 | 0.0206 | 0.0042 | 1.04 x10^-6^ | ++ |
| cg01257889 | *RPH3A* | chr12:113230065 | 0.0333 | 0.0075 | 9.39 x10^-6^ | 0.0467 | 0.0225 | 0.039 | 0.0347 | 0.0071 | 1.09 x10^-6^ | ++ |
| cg20744229 |  | chr11:66154113 | 0.0380 | 0.0078 | 1.23 x10^-6^ | 0.0149 | 0.0137 | 0.28 | 0.0323 | 0.0068 | 1.82 x10^-6^ | ++ |
| **cg19370715** | *CASZ1* | chr1:10710847 | 0.0255 | 0.0059 | 1.92 x10^-5^ | 0.0375 | 0.0171 | 0.029 | 0.0268 | 0.0056 | 1.84 x10^-6^ | ++ |
| **cg18321598** |  | chr12:30684738 | 0.0171 | 0.0040 | 2.51 x10^-5^ | 0.0199 | 0.0092 | 0.030 | 0.0175 | 0.0037 | 2.06 x10^-6^ | ++ |
| **cg16300531** | *KSR2* | chr12:118405988 | 0.0412 | 0.0092 | 7.59 x10^-6^ | 0.0448 | 0.0307 | 0.146 | 0.0415 | 0.0088 | 2.32 x10^-6^ | ++ |
| **cg08698721** | *MEG3* | chr14:101294147 | -0.0148 | 0.0036 | 3.42 x10^-5^ | -0.0222 | 0.0097 | 0.023 | -0.0157 | 0.0033 | 2.70 x10^-6^ | -- |
| **cg03425600** |  | chr12:132648585 | 0.0144 | 0.0035 | 3.89 x10^-5^ | 0.0155 | 0.0071 | 0.029 | 0.0146 | 0.0031 | 2.99 x10^-6^ | ++ |
| **cg18907109** | *HEPACAM2* | chr7:92849682 | -0.0100 | 0.0024 | 2.66 x10^-5^ | -0.0116 | 0.0059 | 0.051 | -0.0102 | 0.0022 | 3.48 x10^-6^ | -- |
| **cg02401554** |  | chr4:57203385 | 0.0124 | 0.0030 | 4.25 x10^-5^ | 0.0167 | 0.0078 | 0.033 | 0.0130 | 0.0028 | 4.18 x10^-6^ | ++ |
| **cg03835140** | *NKPD1* | chr19:45662154 | 0.0132 | 0.0035 | 1.75 x10^-4^ | 0.0436 | 0.0123 | 4.56 x10^-3^ | 0.0155 | 0.0034 | 4.45 x10^-6^ | ++ |
| **cg21931100** |  | chr4:116633269 | -0.0235 | 0.0049 | 2.00 x10^-6^ | -0.0053 | 0.0120 | 0.66 | -0.0208 | 0.0045 | 4.54 x10^-6^ | -- |
| **cg01824138** | *CASZ1* | chr1:10699604 | 0.0349 | 0.0079 | 1.08 x10^-5^ | 0.0273 | 0.0221 | 0.22 | 0.0340 | 0.0074 | 4.75 x10^-6^ | ++ |
| **cg20014974** |  | chr1:8271918 | 0.0154 | 0.0035 | 9.35 x10^-6^ | 0.0098 | 0.0084 | 0.24 | 0.0146 | 0.0032 | 5.14 x10^-6^ | ++ |
| **cg14809891** |  | chr5:5739504 | -0.0219 | 0.0051 | 2.03 x10^-5^ | -0.0203 | 0.0132 | 0.13 | -0.0217 | 0.0048 | 5.53 x10^-6^ | -- |
| **cg03369398** |  | chr12:78973332 | -0.0254 | 0.0057 | 9.71 x10^-6^ | -0.0161 | 0.0144 | 0.27 | -0.0241 | 0.0053 | 5.65 x10^-6^ | -- |
| cg19880864 | *MBNL1-AS1* | chr3:151986749 | -0.0186 | 0.0046 | 4.93 x10^-5^ | -0.0333 | 0.0151 | 0.028 | -0.0199 | 0.0044 | 5.66 x10^-6^ | -- |
| **cg22572362** | *SLC9A3* | chr5:501938 | 0.0260 | 0.0061 | 2.28 x10^-5^ | 0.0261 | 0.0169 | 0.12 | 0.0260 | 0.0057 | 6.09 x10^-6^ | ++ |
| cg16584178 | *NCAM2* | chr21:22611577 | -0.0169 | 0.0042 | 7.04 x10^-5^ | -0.0311 | 0.0132 | 0.019 | -0.0182 | 0.0040 | 6.33 x10^-6^ | -- |
| cg22586324 | *LMOD2* | chr7:123296098 | 0.0182 | 0.0044 | 4.02 x10^-5^ | 0.0213 | 0.0115 | 0.065 | 0.0186 | 0.0041 | 6.51 x10^-6^ | ++ |
| **cg19706390** | *CASZ1* | chr1:10709702 | 0.0354 | 0.0087 | 4.52 x10^-5^ | 0.0488 | 0.0251 | 0.053 | 0.0368 | 0.0082 | 6.72 x10^-6^ | ++ |
| cg26733975 |  | chr7:56551640 | -0.0353 | 0.0081 | 1.38 x10^-5^ | -0.0233 | 0.0185 | 0.21 | -0.0334 | 0.0074 | 6.77 x10^-6^ | -- |
| cg08504812 | *C3orf55* | chr3:157260627 | -0.0222 | 0.0049 | 7.97 x10^-6^ | -0.0110 | 0.0143 | 0.44 | -0.0210 | 0.0047 | 7.19 x10^-6^ | -- |
| **cg16639595** | *SRI* | chr7:87856984 | 0.0145 | 0.0033 | 1.00 x10^-5^ | 0.0318 | 0.0394 | 0.42 | 0.0146 | 0.0033 | 7.28 x10^-6^ | ++ |
| cg16804892 |  | chr14:96241208 | -0.0320 | 0.0079 | 5.27 x10^-5^ | -0.0327 | 0.0171 | 0.058 | -0.0321 | 0.0072 | 7.39 x10^-6^ | -- |
| cg06804344 | *GP9* | chr3:128778699 | -0.0159 | 0.0040 | 8.11 x10^-5^ | -0.0260 | 0.0115 | 0.024 | -0.0170 | 0.0038 | 7.46 x10^-6^ | -- |
| **cg09363850** |  | chr11:58090434 | -0.0294 | 0.0069 | 1.97 x10^-5^ | -0.0393 | 0.0304 | 0.20 | -0.0299 | 0.0067 | 8.06 x10^-6^ | -- |
| cg13063481 |  | chr3:166555550 | -0.0151 | 0.0038 | 7.92 x10^-5^ | -0.0234 | 0.0107 | 0.030 | -0.0160 | 0.0036 | 8.12 x10^-6^ | -- |
| cg20807030 | *MYBL1* | chr8:67525572 | -0.0226 | 0.0053 | 2.43 x10^-5^ | -0.0220 | 0.0158 | 0.16 | -0.0225 | 0.0050 | 8.18 x10^-6^ | -- |
| cg02471243 | *SPATA21* | chr1:16731298 | -0.0165 | 0.0039 | 2.58 x10^-5^ | -0.0146 | 0.0102 | 0.15 | -0.0163 | 0.0037 | 8.38 x10^-6^ | -- |
| **cg00296348** |  | chr10:134721479 | 0.0241 | 0.0056 | 2.00 x10^-5^ | 0.0218 | 0.0182 | 0.23 | 0.0239 | 0.0054 | 8.83 x10^-6^ | ++ |
| cg13983442 | *RFWD2* | chr1:176174365 | 0.0127 | 0.0030 | 2.47 x10^-5^ | 0.0098 | 0.0071 | 0.17 | 0.0123 | 0.0028 | 9.14 x10^-6^ | ++ |
| **cg14534277** | *DSC3* | chr18:28623874 | -0.0200 | 0.0054 | 2.45 x10^-4^ | -0.0349 | 0.0130 | 0.008 | -0.0222 | 0.0050 | 9.66 x10^-6^ | -- |

**Supplementary Table 6:** Results of the sensitivity analyses run in the meta-analysis EWAS sample for the top 36 CpG sites from the original meta-analysis, with % change in effect size (Beta) in the fully-adjusted model compared to the original main model.

| **Probe** | **HG.19 coordinates** | **Beta Main Model** | **P-value Main Model** | **Beta inc. BMI** | **P-value inc. BMI** | **Beta inc. yrs Education** | **P-value inc. yrs Education** | **Beta inc. SIMD** | **P-value inc. SIMD** | **Beta Fully-Adjusted** | **P-value Fully-Adjusted** | **Beta % change from Main Model** |
| --- | --- | --- | --- | --- | --- | --- | --- | --- | --- | --- | --- | --- |
| cg00966482 | chr6:11111926 | 0.021 | 5.97x10^-9^ | 0.021 | 2.52 x10^-8^ | 0.020 | 6.24 x10^-8^ | 0.020 | 2.55 x10^-8^ | 0.019 | 2.90 x10^-7^ | 6.31 |
| cg00590817 | chr1:8272081 | 0.028 | 2.84 x10^-7^ | 0.028 | 1.15 x10^-6^ | 0.029 | 3.80 x10^-7^ | 0.029 | 2.50 x10^-7^ | 0.029 | 2.48 x10^-7^ | -6.16 |
| cg16365064 | chr5:172984486 | 0.021 | 3.24 x10^-7^ | 0.020 | 2.08 x10^-6^ | 0.020 | 4.23 x10^-6^ | 0.020 | 4.07 x10^-6^ | 0.020 | 3.84 x10^-6^ | 2.90 |
| cg27170141 | chr1:10711044 | 0.031 | 4.37 x10^-7^ | 0.029 | 1.28 x10^-5^ | 0.031 | 3.63 x10^-6^ | 0.030 | 7.15 x10^-6^ | 0.030 | 8.94 x10^-6^ | 5.41 |
| cg18321598 | chr12:30684738 | 0.017 | 1.03 x10^-6^ | 0.016 | 1.83 x10^-5^ | 0.016 | 1.71 x10^-5^ | 0.016 | 1.35 x10^-5^ | 0.015 | 7.65 x10^-5^ | 13.79 |
| cg00826892 | chr4:178366013 | 0.031 | 1.34 x10^-6^ | 0.031 | 4.96 x10^-6^ | 0.032 | 2.37 x10^-6^ | 0.029 | 1.41 x10^-5^ | 0.033 | 1.47 x10^-6^ | -5.10 |
| cg26465402 | chr5:7803274 | 0.011 | 1.37 x10^-6^ | 0.010 | 1.20 x10^-5^ | 0.010 | 2.02 x10^-5^ | 0.010 | 9.47 x10^-6^ | 0.010 | 3.77 x10^-5^ | 8.41 |
| cg19370715 | chr1:10710847 | 0.026 | 1.78 x10^-6^ | 0.023 | 8.89 x10^-5^ | 0.025 | 1.91 x10^-5^ | 0.024 | 2.48 x10^-5^ | 0.023 | 7.85 x10^-5^ | 11.54 |
| cg02401554 | chr4:57203385 | 0.013 | 1.87 x10^-6^ | 0.013 | 2.14 x10^-6^ | 0.014 | 8.26 x10^-7^ | 0.014 | 1.17 x10^-6^ | 0.013 | 2.48 x10^-6^ | -3.91 |
| cg08698721 | chr14:101294147 | -0.015 | 2.24 x10^-6^ | -0.015 | 1.52 x10^-5^ | -0.015 | 1.34 x10^-5^ | -0.015 | 1.05 x10^-5^ | -0.014 | 5.68 x10^-5^ | 9.80 |
| cg16639595 | chr7:87856984 | 0.015 | 2.34 x10^-6^ | 0.014 | 1.85 x10^-5^ | 0.015 | 8.01 x10^-6^ | 0.014 | 1.86 x10^-5^ | 0.014 | 1.67 x10^-5^ | 3.38 |
| cg22178513 | chr10:52583805 | -0.034 | 2.86 x10^-6^ | -0.034 | 9.24 x10^-6^ | -0.035 | 3.81 x10^-6^ | -0.032 | 3.32 x10^-5^ | -0.035 | 6.71 x10^-6^ | -2.65 |
| cg16300531 | chr12:118405988 | 0.040 | 3.02 x10^-6^ | 0.040 | 7.84 x10^-6^ | 0.038 | 2.20 x10^-5^ | 0.037 | 2.87 x10^-5^ | 0.041 | 6.98 x10^-6^ | -2.27 |
| cg03369398 | chr12:78973332 | -0.024 | 3.38 x10^-6^ | -0.024 | 1.34 x10^-5^ | -0.024 | 6.88 x10^-6^ | -0.023 | 1.68 x10^-5^ | -0.024 | 1.94 x10^-5^ | 1.67 |
| cg17293641 | chr8:119964144 | 0.020 | 3.41 x10^-6^ | 0.020 | 5.72 x10^-6^ | 0.020 | 8.41 x10^-6^ | 0.020 | 9.47 x10^-6^ | 0.018 | 4.09 x10^-5^ | 8.50 |
| cg03425600 | chr12:132648585 | 0.014 | 3.76 x10^-6^ | 0.015 | 2.21 x10^-6^ | 0.015 | 1.62 x10^-6^ | 0.014 | 4.92 x10^-6^ | 0.016 | 1.47 x10^-6^ | -12.23 |
| cg22572362 | chr5:501938 | 0.026 | 3.84 x10^-6^ | 0.025 | 2.07 x10^-5^ | 0.025 | 2.74 x10^-5^ | 0.025 | 2.09 x10^-5^ | 0.026 | 1.54 x10^-5^ | 0.39 |
| cg14809891 | chr5:5739504 | -0.021 | 4.10 x10^-6^ | -0.022 | 5.77 x10^-6^ | -0.023 | 3.58 x10^-6^ | -0.023 | 3.33 x10^-6^ | -0.022 | 9.09 x10^-6^ | -4.25 |
| cg08490155 | chr14:38676796 | -0.017 | 4.56 x10^-6^ | -0.017 | 1.03 x10^-5^ | -0.017 | 8.43 x10^-6^ | -0.016 | 1.93 x10^-5^ | -0.018 | 8.63 x10^-6^ | -4.79 |
| cg01824138 | chr1:10699604 | 0.033 | 4.65 x10^-6^ | 0.033 | 1.27 x10^-5^ | 0.033 | 1.22 x10^-5^ | 0.033 | 1.20 x10^-5^ | 0.032 | 2.60 x10^-5^ | 1.83 |
| cg14534277 | chr18:28623874 | -0.022 | 5.04 x10^-6^ | -0.022 | 1.07 x10^-5^ | -0.023 | 5.78 x10^-6^ | -0.022 | 8.48 x10^-6^ | -0.022 | 2.44 x10^-5^ | 1.36 |
| cg00296348 | chr10:134721479 | 0.024 | 5.13 x10^-6^ | 0.024 | 8.01 x10^-6^ | 0.023 | 1.96 x10^-5^ | 0.024 | 9.19 x10^-6^ | 0.024 | 1.74 x10^-5^ | 0.42 |
| cg19706390 | chr1:10709702 | 0.036 | 5.47 x10^-6^ | 0.036 | 2.25 x10^-5^ | 0.036 | 1.43 x10^-5^ | 0.036 | 1.93 x10^-5^ | 0.035 | 2.99 x10^-5^ | 2.22 |
| cg18907109 | chr7:92849682 | -0.010 | 5.57 x10^-6^ | -0.009 | 1.58 x10^-5^ | -0.010 | 5.76 x10^-6^ | -0.009 | 2.50 x10^-5^ | -0.009 | 3.53 x10^-5^ | 3.16 |
| cg04988918 | chr4:178366394 | 0.013 | 5.72 x10^-6^ | 0.013 | 1.44 x10^-5^ | 0.014 | 1.29 x10^-5^ | 0.013 | 2.34 x10^-5^ | 0.015 | 3.14 x10^-6^ | -9.02 |
| cg21931100 | chr4:116633269 | -0.020 | 6.35 x10^-6^ | -0.021 | 6.83 x10^-6^ | -0.020 | 1.64 x10^-5^ | -0.019 | 2.36 x10^-5^ | -0.021 | 9.60 x10^-6^ | -4.02 |
| cg24248329 | chr1:41175132 | 0.022 | 6.82 x10^-6^ | 0.019 | 9.52 x10^-5^ | 0.020 | 4.56 x10^-5^ | 0.020 | 3.63 x10^-5^ | 0.019 | 1.04 x10^-4^ | 9.77 |
| cg03835140 | chr19:45662154 | 0.015 | 7.08 x10^-6^ | 0.016 | 6.46 x10^-6^ | 0.015 | 1.50 x10^-5^ | 0.015 | 2.17 x10^-5^ | 0.015 | 2.48 x10^-5^ | 0.68 |
| cg03964940 | chr6:144477779 | 0.022 | 7.24 x10^-6^ | 0.021 | 3.17 x10^-5^ | 0.022 | 1.42 x10^-5^ | 0.022 | 1.83 x10^-5^ | 0.022 | 1.65 x10^-5^ | -2.28 |
| cg01152056 | chr6:166260319 | 0.038 | 7.54 x10^-6^ | 0.037 | 3.15 x10^-5^ | 0.037 | 4.22 x10^-5^ | 0.035 | 1.01 x10^-4^ | 0.036 | 6.88 x10^-5^ | 5.24 |
| cg07593264 | chr22:43410455 | -0.016 | 7.60 x10^-6^ | -0.014 | 1.75 x10^-4^ | -0.015 | 5.23 x10^-5^ | -0.013 | 2.64 x10^-4^ | -0.014 | 2.37 x10^-4^ | 13.46 |
| cg09363850 | chr11:58090434 | -0.029 | 8.03 x10^-6^ | -0.026 | 1.4 x10^-4^ | -0.025 | 2.3 x10^-4^ | -0.024 | 4.5 x10^-4^ | -0.024 | 3.73 x10^-4^ | 15.33 |
| cg03721657 | chr11:1571033 | 0.016 | 8.65 x10^-6^ | 0.015 | 3.48 x10^-5^ | 0.015 | 3.02 x10^-5^ | 0.014 | 1.67 x10^-4^ | 0.014 | 1.29 x10^-4^ | 8.39 |
| cg20014974 | chr1:8271918 | 0.014 | 8.75 x10^-6^ | 0.015 | 4.88 x10^-6^ | 0.015 | 3.84 x10^-6^ | 0.015 | 5.59 x10^-6^ | 0.016 | 3.27 x10^-6^ | -11.51 |
| cg26086468 | chr18:5628160 | -0.014 | 9.90 x10^-6^ | -0.014 | 3.75 x10^-5^ | -0.013 | 4.10 x10^-5^ | -0.014 | 3.07 x10^-5^ | -0.013 | 6.16 x10^-5^ | 3.62 |
| cg19863411 | chr17:79869801 | 0.022 | 9.98 x10^-6^ | 0.019 | 2.85 x10^-4^ | 0.019 | 2.28 x10^-4^ | 0.020 | 1.38 x10^-4^ | 0.020 | 1.85 x10^-4^ | 9.63 |

**Supplementary Table 7:** Significant (p<5x10^-8^) GWAS catalogue outputs for SNPs in genes that mapped to the CpG sites from the main meta-analysis EWAS with p<1x10^-5^.

| Gene | Trait | SNP | | P-Value | Study Link |
| --- | --- | --- | --- | --- | --- |
| *A1CF* | Glomerular filtration rate in non diabetics (creatinine) | | rs10994860 | 1.00E-12 | www.ncbi.nlm.nih.gov/pubmed/26831199 |
| *A1CF* | Glomerular filtration rate (creatinine) | | rs10994856 | 5.00E-09 | www.ncbi.nlm.nih.gov/pubmed/28452372 |
| *A1CF* | Estimated glomerular filtration rate | rs10994860 | | 2.00E-24 | www.ncbi.nlm.nih.gov/pubmed/31152163 |
| *A1CF* | Estimated glomerular filtration rate | rs10994856 | | 1.00E-14 | www.ncbi.nlm.nih.gov/pubmed/31451708 |
| *A1CF* | Urate levels | rs10994860 | | 1.00E-36 | www.ncbi.nlm.nih.gov/pubmed/31578528 |
| *A1CF* | Urate levels | rs10994860 | | 4.00E-29 | www.ncbi.nlm.nih.gov/pubmed/31578528 |
| *A1CF* | Urate levels | rs10994860 | | 7.00E-42 | www.ncbi.nlm.nih.gov/pubmed/31578528 |
| *A1CF* | Serum uric acid levels | rs10994856 | | 3.00E-13 | www.ncbi.nlm.nih.gov/pubmed/30993211 |
| *A1CF* | Estimated glomerular filtration rate | rs10994856 | | 1.00E-10 | www.ncbi.nlm.nih.gov/pubmed/31015462 |
| *A1CF* | Urate levels | rs10994860 | | 2.00E-12 | www.ncbi.nlm.nih.gov/pubmed/31578528 |
| *A1CF* | Core binding factor acute myeloid leukemia | Haplotype block | | 1.00E-15 | www.ncbi.nlm.nih.gov/pubmed/27903959 |
| *A1CF* | Core binding factor acute myeloid leukemia | Haplotype block | | 5.00E-11 | www.ncbi.nlm.nih.gov/pubmed/27903959 |
| *A1CF - CCDC58P2* | Urate levels | rs10821905 | | 7.00E-17 | www.ncbi.nlm.nih.gov/pubmed/23263486 |
| *A1CF - CCDC58P2* | Estimated glomerular filtration rate | rs10821905 | | 5.00E-26 | www.ncbi.nlm.nih.gov/pubmed/31152163 |
| *A1CF - CCDC58P2* | Colorectal cancer or advanced adenoma | rs10821907 | | 5.00E-10 | www.ncbi.nlm.nih.gov/pubmed/30510241 |
| *A1CF - CCDC58P2* | Estimated glomerular filtration rate in non-diabetics | rs10821907 | | 1.00E-08 | www.ncbi.nlm.nih.gov/pubmed/31451708 |
| *A1CF - CCDC58P2* | Red blood cell count | rs10821907 | | 3.00E-08 | www.ncbi.nlm.nih.gov/pubmed/30595370 |
| *A1CF, ASAH2B* | Triglyceride levels x alcohol consumption (regular vs non-regular drinkers) interaction (2df) | rs41274050 | | 4.00E-09 | www.ncbi.nlm.nih.gov/pubmed/30698716 |
| *A1CF, ASAH2B* | Triglyceride levels | rs41274050 | | 5.00E-19 | www.ncbi.nlm.nih.gov/pubmed/29083408 |
| *A1CF, ASAH2B* | Triglyceride levels | rs41274050 | | 4.00E-09 | www.ncbi.nlm.nih.gov/pubmed/29083408 |
| *A1CF, ASAH2B* | Triglyceride levels x alcohol consumption (regular vs non-regular drinkers) interaction (2df) | rs41274050 | | 1.00E-09 | www.ncbi.nlm.nih.gov/pubmed/30698716 |
| *A1CF, ASAH2B* | Total cholesterol levels | rs41274050 | | 6.00E-13 | www.ncbi.nlm.nih.gov/pubmed/30275531 |
| *A1CF, ASAH2B* | LDL cholesterol | rs41274050 | | 6.00E-10 | www.ncbi.nlm.nih.gov/pubmed/30275531 |
| *A1CF, ASAH2B* | Triglycerides | rs41274050 | | 4.00E-09 | www.ncbi.nlm.nih.gov/pubmed/30275531 |
| *ADCY2* | Cognitive decline rate in late mild cognitive impairment | rs78277363 | | 3.00E-08 | www.ncbi.nlm.nih.gov/pubmed/26252872 |
| *ADCY2* | Bipolar disorder | rs17826816 | | 1.00E-08 | www.ncbi.nlm.nih.gov/pubmed/24618891 |
| *ADCY2* | Bipolar disorder (age of onset <21) or attention deficit hyperactivity disorder | rs58502974 | | 2.00E-08 | www.ncbi.nlm.nih.gov/pubmed/27890468 |
| *ADCY2* | Intelligence | rs17826816 | | 2.00E-08 | www.ncbi.nlm.nih.gov/pubmed/29942086 |
| *ADCY2* | Adolescent idiopathic scoliosis | rs7704053 | | 7.00E-27 | www.ncbi.nlm.nih.gov/pubmed/30019117 |
| *ADCY2* | Lung function (FEV1/FVC) | rs13166360 | | 7.00E-09 | www.ncbi.nlm.nih.gov/pubmed/30595370 |
| *ADCY2* | Highest math class taken (MTAG) | rs78765360 | | 2.00E-14 | www.ncbi.nlm.nih.gov/pubmed/30038396 |
| *ADCY2* | Cognitive performance (MTAG) | rs78765360 | | 1.00E-09 | www.ncbi.nlm.nih.gov/pubmed/30038396 |
| *ADCY2* | Self-reported math ability (MTAG) | rs78765360 | | 2.00E-14 | www.ncbi.nlm.nih.gov/pubmed/30038396 |
| *ADCY2* | Bipolar disorder | rs200550695 | | 1.00E-08 | www.ncbi.nlm.nih.gov/pubmed/31043756 |
| *ADCY2* | Bipolar I disorder | rs200550695 | | 7.00E-09 | www.ncbi.nlm.nih.gov/pubmed/31043756 |
| *LINC02142 - ADCY2* | Educational attainment (MTAG) | rs12515392 | | 4.00E-10 | www.ncbi.nlm.nih.gov/pubmed/30038396 |
| *LINC02142* | Coffee consumption | rs993885 | | 2.00E-09 | www.ncbi.nlm.nih.gov/pubmed/31046077 |
| *LINC02142* | Plasma factor VII activating protease levels | rs35510613 | | 1.00E-08 | www.ncbi.nlm.nih.gov/pubmed/30070759 |
| *APOC1 - APOC1P1* | Alzheimer's disease or HDL levels (pleiotropy) | rs157595 | | 1.00E-97 | www.ncbi.nlm.nih.gov/pubmed/30805717 |
| *CASZ1* | Blood pressure | rs880315 | | 3.00E-10 | www.ncbi.nlm.nih.gov/pubmed/21572416 |
| *CASZ1* | Hypertension | rs880315 | | 2.00E-09 | www.ncbi.nlm.nih.gov/pubmed/25249183 |
| *CASZ1* | Systolic blood pressure | rs880315 | | 6.00E-10 | www.ncbi.nlm.nih.gov/pubmed/25249183 |
| *CASZ1* | Systolic blood pressure (cigarette smoking interaction) | rs880315 | | 2.00E-54 | www.ncbi.nlm.nih.gov/pubmed/29455858 |
| *CASZ1* | Diastolic blood pressure | rs880315 | | 4.00E-10 | www.ncbi.nlm.nih.gov/pubmed/28739976 |
| *CASZ1* | Systolic blood pressure | rs880315 | | 9.00E-16 | www.ncbi.nlm.nih.gov/pubmed/28739976 |
| *CASZ1* | Pulse pressure | rs880315 | | 2.00E-10 | www.ncbi.nlm.nih.gov/pubmed/28739976 |
| *CASZ1* | Diastolic blood pressure (cigarette smoking interaction) | rs880315 | | 7.00E-42 | www.ncbi.nlm.nih.gov/pubmed/29455858 |
| *CASZ1* | Diastolic blood pressure | rs12046278 | | 2.00E-12 | www.ncbi.nlm.nih.gov/pubmed/27841878 |
| *CASZ1* | Diastolic blood pressure | rs880315 | | 2.00E-12 | www.ncbi.nlm.nih.gov/pubmed/27841878 |
| *CASZ1* | Systolic blood pressure | rs12046278 | | 5.00E-18 | www.ncbi.nlm.nih.gov/pubmed/27841878 |
| *CASZ1* | Pulse pressure | rs880315 | | 6.00E-09 | www.ncbi.nlm.nih.gov/pubmed/27841878 |
| *CASZ1* | Diastolic blood pressure | rs880315 | | 1.00E-11 | www.ncbi.nlm.nih.gov/pubmed/27618452 |
| *CASZ1* | Systolic blood pressure | rs12046278 | | 1.00E-08 | www.ncbi.nlm.nih.gov/pubmed/27841878 |
| *CASZ1* | Systolic blood pressure | rs880315 | | 2.00E-14 | www.ncbi.nlm.nih.gov/pubmed/27618452 |
| *CASZ1* | Pulse pressure | rs12046278 | | 3.00E-09 | www.ncbi.nlm.nih.gov/pubmed/27841878 |
| *CASZ1* | Systolic blood pressure | rs880315 | | 5.00E-17 | www.ncbi.nlm.nih.gov/pubmed/27841878 |
| *CASZ1* | Diastolic blood pressure | rs12046278 | | 7.00E-09 | www.ncbi.nlm.nih.gov/pubmed/27841878 |
| *CASZ1* | Urinary albumin-to-creatinine ratio | rs17035646 | | 1.00E-08 | www.ncbi.nlm.nih.gov/pubmed/31511532 |
| *CASZ1* | Resistant hypertension | rs12046278 | | 2.00E-09 | www.ncbi.nlm.nih.gov/pubmed/31545351 |
| *CASZ1* | Resistant hypertension | rs12046278 | | 1.00E-08 | www.ncbi.nlm.nih.gov/pubmed/31545351 |
| *CASZ1* | Resistance to antihypertensive treatment in hypertension | rs12046278 | | 9.00E-09 | www.ncbi.nlm.nih.gov/pubmed/31545351 |
| *CASZ1* | Urinary albumin-to-creatinine ratio | rs34071855 | | 7.00E-10 | www.ncbi.nlm.nih.gov/pubmed/31511532 |
| *CASZ1* | Microalbuminuria | rs34071855 | | 6.00E-09 | www.ncbi.nlm.nih.gov/pubmed/31511532 |
| *CASZ1* | Mean arterial pressure | rs880315 | | 5.00E-17 | www.ncbi.nlm.nih.gov/pubmed/27618448 |
| *CASZ1* | Pulse pressure | rs34071855 | | 2.00E-10 | www.ncbi.nlm.nih.gov/pubmed/30578418 |
| *CASZ1* | Male-pattern baldness | rs61776295 | | 2.00E-14 | www.ncbi.nlm.nih.gov/pubmed/30573740 |
| *CASZ1* | Systolic blood pressure | rs17035646 | | 2.00E-33 | www.ncbi.nlm.nih.gov/pubmed/30578418 |
| *CASZ1* | Male-pattern baldness | rs143353512 | | 2.00E-42 | www.ncbi.nlm.nih.gov/pubmed/30573740 |
| *CASZ1* | Appendicular lean mass | rs11121615 | | 2.00E-13 | www.ncbi.nlm.nih.gov/pubmed/31761296 |
| *CASZ1* | Facial morphology | rs143353512 | | 6.00E-09 | www.ncbi.nlm.nih.gov/pubmed/31763980 |
| *CASZ1* | Pulse pressure | rs880315 | | 5.00E-09 | www.ncbi.nlm.nih.gov/pubmed/29403010 |
| *CASZ1* | Mean arterial pressure | rs880315 | | 2.00E-16 | www.ncbi.nlm.nih.gov/pubmed/29403010 |
| *CASZ1* | Systolic blood pressure | rs880315 | | 7.00E-18 | www.ncbi.nlm.nih.gov/pubmed/29403010 |
| *CASZ1* | Diastolic blood pressure | rs880315 | | 6.00E-12 | www.ncbi.nlm.nih.gov/pubmed/29403010 |
| *CASZ1* | Diastolic blood pressure | rs880315 | | 6.00E-16 | www.ncbi.nlm.nih.gov/pubmed/27618447 |
| *CASZ1* | Balding type 1 | rs143353512 | | 6.00E-25 | www.ncbi.nlm.nih.gov/pubmed/30595370 |
| *CASZ1* | Systolic blood pressure | rs880315 | | 1.00E-23 | www.ncbi.nlm.nih.gov/pubmed/30487518 |
| *CASZ1* | Pulse pressure | rs880315 | | 8.00E-12 | www.ncbi.nlm.nih.gov/pubmed/30487518 |
| *CASZ1* | Hypertension | rs880315 | | 7.00E-24 | www.ncbi.nlm.nih.gov/pubmed/30487518 |
| *CASZ1* | Diastolic blood pressure | rs880315 | | 1.00E-18 | www.ncbi.nlm.nih.gov/pubmed/30487518 |
| *CASZ1* | Mean arterial pressure | rs880315 | | 3.00E-23 | www.ncbi.nlm.nih.gov/pubmed/30487518 |
| *CASZ1* | Ischemic stroke | rs880315 | | 6.00E-09 | www.ncbi.nlm.nih.gov/pubmed/29531354 |
| *CASZ1* | Systolic blood pressure x alcohol consumption (light vs heavy) interaction (2df test) | rs17035646 | | 7.00E-14 | www.ncbi.nlm.nih.gov/pubmed/29912962 |
| *CASZ1* | Systolic blood pressure x alcohol consumption (light vs heavy) interaction (2df test) | rs17035646 | | 9.00E-13 | www.ncbi.nlm.nih.gov/pubmed/29912962 |
| *CASZ1* | Systolic blood pressure x alcohol consumption interaction (2df test) | rs35295665 | | 5.00E-27 | www.ncbi.nlm.nih.gov/pubmed/29912962 |
| *CASZ1* | Diastolic blood pressure x alcohol consumption interaction (2df test) | rs34071855 | | 5.00E-29 | www.ncbi.nlm.nih.gov/pubmed/29912962 |
| *CASZ1* | Body mass index | rs284262 | | 7.00E-09 | www.ncbi.nlm.nih.gov/pubmed/30595370 |
| *CASZ1* | Systolic blood pressure x alcohol consumption interaction (2df test) | rs17035646 | | 1.00E-36 | www.ncbi.nlm.nih.gov/pubmed/29912962 |
| *CASZ1* | Diastolic blood pressure x alcohol consumption (light vs heavy) interaction (2df test) | rs17035646 | | 1.00E-11 | www.ncbi.nlm.nih.gov/pubmed/29912962 |
| *CASZ1* | Mean arterial pressure x alcohol consumption (light vs heavy) interaction (2df test) | rs17035646 | | 5.00E-14 | www.ncbi.nlm.nih.gov/pubmed/29912962 |
| *CASZ1* | Mean arterial pressure x alcohol consumption interaction (2df test) | rs34071855 | | 4.00E-20 | www.ncbi.nlm.nih.gov/pubmed/29912962 |
| *CASZ1* | Pulse pressure x alcohol consumption interaction (2df test) | rs17035646 | | 2.00E-20 | www.ncbi.nlm.nih.gov/pubmed/29912962 |
| *CASZ1* | Pulse pressure x alcohol consumption interaction (2df test) | rs35295665 | | 9.00E-17 | www.ncbi.nlm.nih.gov/pubmed/29912962 |
| *CASZ1* | Diastolic blood pressure x alcohol consumption interaction (2df test) | rs34071855 | | 2.00E-26 | www.ncbi.nlm.nih.gov/pubmed/29912962 |
| *CASZ1* | Body mass index | rs544722 | | 3.00E-09 | www.ncbi.nlm.nih.gov/pubmed/30239722 |
| *CASZ1* | Atrial fibrillation | rs880315 | | 5.00E-09 | www.ncbi.nlm.nih.gov/pubmed/29892015 |
| *CASZ1* | Estimated glomerular filtration rate | rs74748843 | | 3.00E-10 | www.ncbi.nlm.nih.gov/pubmed/31152163 |
| *CASZ1* | Stroke | rs880315 | | 4.00E-10 | www.ncbi.nlm.nih.gov/pubmed/29531354 |
| *CASZ1* | Male-pattern baldness | rs59304342 | | 4.00E-08 | www.ncbi.nlm.nih.gov/pubmed/28196072 |
| *CASZ1* | Medication use (calcium channel blockers) | rs880315 | | 1.00E-30 | www.ncbi.nlm.nih.gov/pubmed/31015401 |
| *CASZ1* | Varicose veins | rs11121615 | | 3.00E-76 | www.ncbi.nlm.nih.gov/pubmed/30998689 |
| *CASZ1* | Medication use (agents acting on the renin-angiotensin system) | rs880315 | | 7.00E-24 | www.ncbi.nlm.nih.gov/pubmed/31015401 |
| *CASZ1* | Estimated glomerular filtration rate | rs284316 | | 2.00E-09 | www.ncbi.nlm.nih.gov/pubmed/31015462 |
| *CASZ1* | Medication use (beta blocking agents) | rs12046278 | | 3.00E-13 | www.ncbi.nlm.nih.gov/pubmed/31015401 |
| *CASZ1* | Medication use (antihypertensives) | rs34071855 | | 7.00E-09 | www.ncbi.nlm.nih.gov/pubmed/31015401 |
| *CASZ1* | Medication use (diuretics) | rs880315 | | 2.00E-26 | www.ncbi.nlm.nih.gov/pubmed/31015401 |
| *CASZ1* | Varicose veins | rs11121615 | | 4.00E-65 | www.ncbi.nlm.nih.gov/pubmed/30566020 |
| *CASZ1* | Chronic obstructive pulmonary disease or resting heart rate (pleiotropy) | rs59985166 | | 4.00E-18 | www.ncbi.nlm.nih.gov/pubmed/30940143 |
| *CASZ1* | Body mass index | rs284262 | | 5.00E-09 | www.ncbi.nlm.nih.gov/pubmed/31669095 |
| *CASZ1* | Atrial fibrillation | rs284277 | | 1.00E-09 | www.ncbi.nlm.nih.gov/pubmed/30061737 |
| *CASZ1* | White blood cell count | rs284316 | | 1.00E-08 | www.ncbi.nlm.nih.gov/pubmed/30595370 |
| *CASZ1* | Cardiovascular disease | rs880315 | | 2.00E-24 | www.ncbi.nlm.nih.gov/pubmed/30595370 |
| *CASZ1* | Systolic blood pressure | rs17035646 | | 4.00E-36 | www.ncbi.nlm.nih.gov/pubmed/30595370 |
| *COLEC10* | Bone mineral density (hip) | rs11995824 | | 7.00E-09 | www.ncbi.nlm.nih.gov/pubmed/19801982 |
| *COLEC10* | Bone mineral density | rs4424296 | | 9.00E-14 | www.ncbi.nlm.nih.gov/pubmed/24249740 |
| *COLEC10* | Bone mineral density (paediatric, skull) | rs2450083 | | 2.00E-11 | www.ncbi.nlm.nih.gov/pubmed/24945404 |
| *COLEC10* | Bone mineral density (paediatric, skull) | rs2450083 | | 3.00E-08 | www.ncbi.nlm.nih.gov/pubmed/24945404 |
| *COLEC10* | Lumbar spine bone mineral density | rs13264172 | | 3.00E-25 | www.ncbi.nlm.nih.gov/pubmed/29499414 |
| *COLEC10* | Femoral neck bone mineral density | rs13264172 | | 1.00E-16 | www.ncbi.nlm.nih.gov/pubmed/29499414 |
| *COLEC10* | Asthma (childhood onset) | rs2450083 | | 8.00E-11 | www.ncbi.nlm.nih.gov/pubmed/30929738 |
| *COLEC10 - MAL2* | Blood protein levels in cardiovascular risk | rs7813952 | | 1.00E-21 | www.ncbi.nlm.nih.gov/pubmed/28369058 |
| *COLEC10 - MAL2* | Blood protein levels in cardiovascular risk | rs6469811 | | 3.00E-13 | www.ncbi.nlm.nih.gov/pubmed/28369058 |
| *AC107953.1 - COLEC10* | Bone mineral density (spine) | rs2062377 | | 4.00E-16 | www.ncbi.nlm.nih.gov/pubmed/19801982 |
| *AC107953.1 - COLEC10* | Lumbar spine bone mineral density | rs2062377 | | 3.00E-39 | www.ncbi.nlm.nih.gov/pubmed/22504420 |
| *KHDRBS3 x KSR2* | Monoclonal gammopathy of undetermined significance | rs4909494 x rs10774941 | | 4.00E-10 | www.ncbi.nlm.nih.gov/pubmed/30134812 |
| *KSR2* | Depressive symptoms (MTAG) | rs7134419 | | 4.00E-08 | www.ncbi.nlm.nih.gov/pubmed/29292387 |
| *KSR2* | Depression | rs7973260 | | 2.00E-08 | www.ncbi.nlm.nih.gov/pubmed/27089181 |
| *KSR2* | Coronary artery disease | rs11830157 | | 2.00E-09 | www.ncbi.nlm.nih.gov/pubmed/26343387 |
| *KSR2* | Post bronchodilator FEV1/FVC ratio | rs183032784 | | 6.00E-09 | www.ncbi.nlm.nih.gov/pubmed/26634245 |
| *KSR2* | Body mass index | rs9805009 | | 4.00E-09 | www.ncbi.nlm.nih.gov/pubmed/30595370 |
| *KSR2* | Male-pattern baldness | rs61640416 | | 4.00E-09 | www.ncbi.nlm.nih.gov/pubmed/30573740 |
| *KSR2* | Insomnia | rs4767645 | | 6.00E-10 | www.ncbi.nlm.nih.gov/pubmed/30804565 |
| *KSR2* | Sleep duration | rs4767550 | | 3.00E-09 | www.ncbi.nlm.nih.gov/pubmed/30804565 |
| *KSR2* | Body mass index | rs56214831 | | 1.00E-08 | www.ncbi.nlm.nih.gov/pubmed/29273807 |
| *KSR2* | Body mass index | rs9805009 | | 7.00E-09 | www.ncbi.nlm.nih.gov/pubmed/31669095 |
| *KSR2* | Type 2 diabetes | rs79310463 | | 2.00E-08 | www.ncbi.nlm.nih.gov/pubmed/30718926 |
| *KSR2* | Sleep duration | rs4767550 | | 1.00E-10 | www.ncbi.nlm.nih.gov/pubmed/30846698 |
| *KSR2* | Lung function (FEV1/FVC) | rs12424627 | | 4.00E-10 | www.ncbi.nlm.nih.gov/pubmed/30595370 |
| *KSR2 - RFC5* | Body mass index | rs7963783 | | 7.00E-10 | www.ncbi.nlm.nih.gov/pubmed/30239722 |
| *KSR2 - RFC5* | Type 2 diabetes | rs34965774 | | 2.00E-09 | www.ncbi.nlm.nih.gov/pubmed/30297969 |
| *KSR2 - RFC5* | Body mass index | rs7968390 | | 2.00E-10 | www.ncbi.nlm.nih.gov/pubmed/30239722 |
| *MAL2* | Osteoprotegerin levels | rs1425053 | | 8.00E-15 | www.ncbi.nlm.nih.gov/pubmed/25080503 |
| *MAL2* | Osteoprotegerin levels | rs2468186 | | 8.00E-09 | www.ncbi.nlm.nih.gov/pubmed/25080503 |
| *MARK4, NKPD1* | Alzheimer's disease or family history of Alzheimer's disease | rs10421247 | | 7.00E-09 | www.ncbi.nlm.nih.gov/pubmed/30617256 |
| *MARK4, PPP1R37* | Cerebrospinal fluid AB1-42 levels | rs149151450 | | 6.00E-10 | www.ncbi.nlm.nih.gov/pubmed/28247064 |
| *NKPD1, MARK4* | Alzheimer's disease or family history of Alzheimer's disease | rs28469095 | | 2.00E-38 | www.ncbi.nlm.nih.gov/pubmed/30617256 |
| *DLK1 - MEG3* | Diastolic blood pressure response to thiazide and thiazide-like diuretics in hypertension | rs2400940 | | 4.00E-08 | www.ncbi.nlm.nih.gov/pubmed/31754133 |
| *MEG3* | Birth weight | rs6575803 | | 1.00E-12 | www.ncbi.nlm.nih.gov/pubmed/31043758 |
| *MEG3* | Type 1 diabetes | rs941576 | | 1.00E-10 | www.ncbi.nlm.nih.gov/pubmed/19966805 |
| *MEG3* | Type 1 diabetes | rs56994090 | | 1.00E-11 | www.ncbi.nlm.nih.gov/pubmed/25751624 |
| *MIR4456 - AC106772.1* | Ulcerative colitis | rs11739663 | | 2.00E-08 | www.ncbi.nlm.nih.gov/pubmed/23128233 |
| *MIR4456 - AC106772.1* | Lung disease severity in cystic fibrosis | rs57221529 | | 1.00E-11 | www.ncbi.nlm.nih.gov/pubmed/26417704 |
| *MIR4456 - AC106772.1* | Lung disease severity in cystic fibrosis | rs57221529 | | 7.00E-12 | www.ncbi.nlm.nih.gov/pubmed/26417704 |
| *NECTIN2* | Alzheimer's disease or HDL levels (pleiotropy) | rs41290120 | | 3.00E-36 | www.ncbi.nlm.nih.gov/pubmed/30805717 |
| *PEX14* | Male-pattern baldness | rs2242288 | | 3.00E-10 | www.ncbi.nlm.nih.gov/pubmed/28196072 |
| *PRKG1* | Interferon alpha levels in systemic lupus erythematosus | rs7897633 | | 3.00E-08 | www.ncbi.nlm.nih.gov/pubmed/25338677 |
| *SAMD12-AS1* | Heel bone mineral density | rs117108011 | | 1.00E-13 | www.ncbi.nlm.nih.gov/pubmed/28869591 |
| *SAMD12-AS1* | Heel bone mineral density | rs1353171 | | 7.00E-15 | www.ncbi.nlm.nih.gov/pubmed/30598549 |
| *SAMD12-AS1* | Heel bone mineral density | rs117204589 | | 6.00E-35 | www.ncbi.nlm.nih.gov/pubmed/30598549 |
| *SAMD12-AS1* | Heel bone mineral density | rs117108011 | | 5.00E-11 | www.ncbi.nlm.nih.gov/pubmed/28869591 |
| *SAMD12-AS1 - TNFRSF11B* | Bone mineral density | rs4355801 | | 8.00E-10 | www.ncbi.nlm.nih.gov/pubmed/18455228 |
| *SAMD12-AS1 - TNFRSF11B* | Heel bone mineral density | rs7813157 | | 5.00E-22 | www.ncbi.nlm.nih.gov/pubmed/30048462 |
| *TNFRSF11B* | Eosinophil counts | rs7834745 | | 9.00E-10 | www.ncbi.nlm.nih.gov/pubmed/27863252 |
| *TNFRSF11B* | Sum eosinophil basophil counts | rs7834745 | | 7.00E-09 | www.ncbi.nlm.nih.gov/pubmed/27863252 |
| *TNFRSF11B* | Serum alkaline phosphatase levels | rs11573824 | | 2.00E-22 | www.ncbi.nlm.nih.gov/pubmed/29403010 |
| *TNFRSF11B* | Total body bone mineral density (age 45-60) | rs7010267 | | 3.00E-15 | www.ncbi.nlm.nih.gov/pubmed/29304378 |
| *TNFRSF11B* | Medication use (thyroid preparations) | rs1032129 | | 6.00E-10 | www.ncbi.nlm.nih.gov/pubmed/31015401 |
| *TNFRSF11B* | Hypothyroidism | rs1032129 | | 3.00E-10 | www.ncbi.nlm.nih.gov/pubmed/30595370 |
| *TNFRSF11B - RNU6-12P* | Bone mineral density | rs7839059 | | 1.00E-10 | www.ncbi.nlm.nih.gov/pubmed/23437003 |
| *TNFRSF11B - RNU6-12P* | Osteoporosis-related phenotypes | rs2062375 | | 3.00E-11 | www.ncbi.nlm.nih.gov/pubmed/20548944 |
| *TNFRSF11B - RNU6-12P* | Bone mineral density (hip) | rs7839059 | | 7.00E-09 | www.ncbi.nlm.nih.gov/pubmed/27311723 |
| *TNFRSF11B - RNU6-12P* | Heel bone mineral density | rs1385504 | | 4.00E-12 | www.ncbi.nlm.nih.gov/pubmed/30598549 |
| *TNFRSF11B - RNU6-12P* | Serum alkaline phosphatase levels | rs4242592 | | 3.00E-10 | www.ncbi.nlm.nih.gov/pubmed/31666285 |
| *TNFRSF11B - RNU6-12P* | Waist circumference adjusted for body mass index | rs1385499 | | 4.00E-10 | www.ncbi.nlm.nih.gov/pubmed/31669095 |
| *TNFRSF11B - RNU6-12P* | Lumbar spine bone mineral density (trabecular) | rs1485303 | | 5.00E-09 | www.ncbi.nlm.nih.gov/pubmed/27476799 |
| *TNFRSF11B - RNU6-12P* | Eosinophil counts | rs12681644 | | 4.00E-10 | www.ncbi.nlm.nih.gov/pubmed/30595370 |
| *SLC9A3* | Lung disease severity in cystic fibrosis | rs56302516 | | 8.00E-10 | www.ncbi.nlm.nih.gov/pubmed/26417704 |
| *SLC9A3* | Lung disease severity in cystic fibrosis | rs56302516 | | 4.00E-09 | www.ncbi.nlm.nih.gov/pubmed/26417704 |
| *SLC9A3* | Estimated glomerular filtration rate | rs6555317 | | 2.00E-09 | www.ncbi.nlm.nih.gov/pubmed/31152163 |
| *STX11* | Pre-treatment viral load in HIV-1 infection | rs7755842 | | 2.00E-17 | www.ncbi.nlm.nih.gov/pubmed/31219150 |
| *AC011481.1* | Alzheimer's disease or HDL levels (pleiotropy) | rs7254723 | | 7.00E-22 | www.ncbi.nlm.nih.gov/pubmed/30805717 |
| *AC090506.2 - AC016382.1* | Supraventricular ectopy | rs8086068 | | 3.00E-09 | www.ncbi.nlm.nih.gov/pubmed/29618737 |
| *AC115100.1 - AC090506.1* | Metabolite levels | rs11083411 | | 9.00E-09 | www.ncbi.nlm.nih.gov/pubmed/31628463 |
